# Induced-volatolomics for the design of tumour activated therapy†

**DOI:** 10.1039/d2sc06797h

**Published:** 2023-04-11

**Authors:** Rémi Châtre, Estelle Blochouse, Rony Eid, Fabiola Djago, Justin Lange, Mehrad Tarighi, Brigitte Renoux, Julien Sobilo, Alain Le Pape, Jonathan Clarhaut, Claude Geffroy, Isabelle Opalinski, Wei Tuo, Sébastien Papot, Pauline Poinot

**Affiliations:** a University of Poitiers, UMR CNRS 7285, Institut de Chimie des Milieux et Matériaux de Poitiers (IC2MP), Equipe Labellisée Ligue Contre le Cancer 4 Rue Michel-Brunet, TSA 51106 86073 Poitiers Cedex 9 France pauline.poinot@univ-poitiers.fr sebastien.papot@univ-poitiers.fr; b UAR No. 44 PHENOMIN TAAM-Imagerie In Vivo, CNRS 3B Rue de la Férollerie F-45071 Orléans France; c CHU de Poitiers 2 Rue de la Miléterie, CS 90577 F-86021 Poitiers France; d Seekyo SA 2 Avenue Galilée, BP 30153 86961 Futuroscope France

## Abstract

The discovery of tumour-associated markers is of major interest for the development of selective cancer chemotherapy. Within this framework, we introduced the concept of induced-volatolomics enabling to monitor simultaneously the dysregulation of several tumour-associated enzymes in living mice or biopsies. This approach relies on the use of a cocktail of volatile organic compound (VOC)-based probes that are activated enzymatically for releasing the corresponding VOCs. Exogenous VOCs can then be detected in the breath of mice or in the headspace above solid biopsies as specific tracers of enzyme activities. Our induced-volatolomics modality highlighted that the up-regulation of *N*-acetylglucosaminidase was a hallmark of several solid tumours. Having identified this glycosidase as a potential target for cancer therapy, we designed an enzyme-responsive albumin-binding prodrug of the potent monomethyl auristatin E programmed for the selective release of the drug in the tumour microenvironment. This tumour activated therapy produced a remarkable therapeutic efficacy on orthotopic triple-negative mammary xenografts in mice, leading to the disappearance of tumours in 66% of treated animals. Thus, this study shows the potential of induced-volatolomics for the exploration of biological processes as well as the discovery of novel therapeutic strategies.

## Abstract 2

We developed a volatile organic compound (VOC)-based probe cocktail for monitoring the dysregulation of tumour-associated enzymes *via* the detection of volatile tracers in animal breath, thereby allowing the discovery of a new tumour targeted therapy.

## Graphical abstract

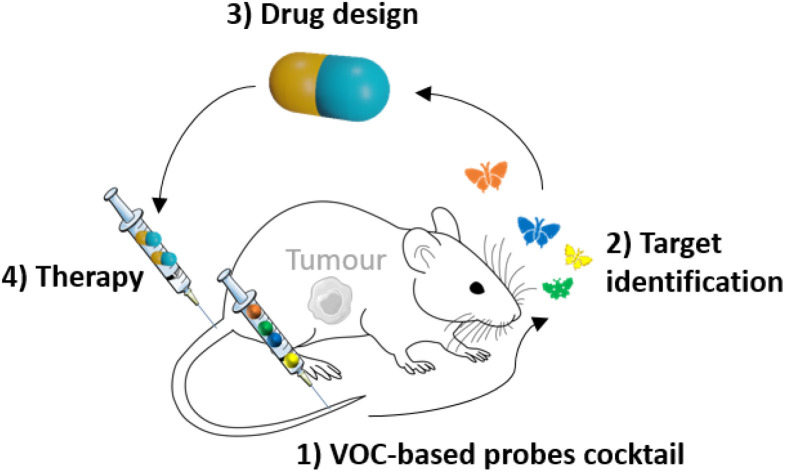

Enzymes are major targets for drug discovery in oncology.^1,2^ The current therapeutic arsenal includes numerous enzyme inhibitors^3^ and enzyme-responsive antibody–drug conjugates^4^ for the treatment of a wide range of malignancies. Thus, the design of novel strategies dedicated to the exploration of tumour-associated enzymes is of prime importance for cancer therapy and diagnosis.^5,6^ Within this framework, activable imaging probes have been developed to detect the dysregulation of some enzyme activities in tumours.^7–17^ However, most of these turn-on probes suffer from low signal penetration depth and background noise.^18,19^ Recently, we introduced the concept of induced-volatolomics for cancer diagnosis.^20,21^ We demonstrated that a volatile organic compound (VOC)-based probe can be activated *in vivo* by an enzyme overexpressed in solid tumours to trigger the release of an exogenous volatile tracer in the breath of individuals, thereby enabling cancer detection. Herein, we present an original approach allowing real time and simultaneous monitoring of the activity of several tumour-associated enzymes by means of a VOC-based probe cocktail (Fig. 1). This induced-volatolomics modality allowed the identification of upregulated enzymes within the tumour, leading to the development of a new enzyme-targeted drug candidate.

**Fig. 1:**
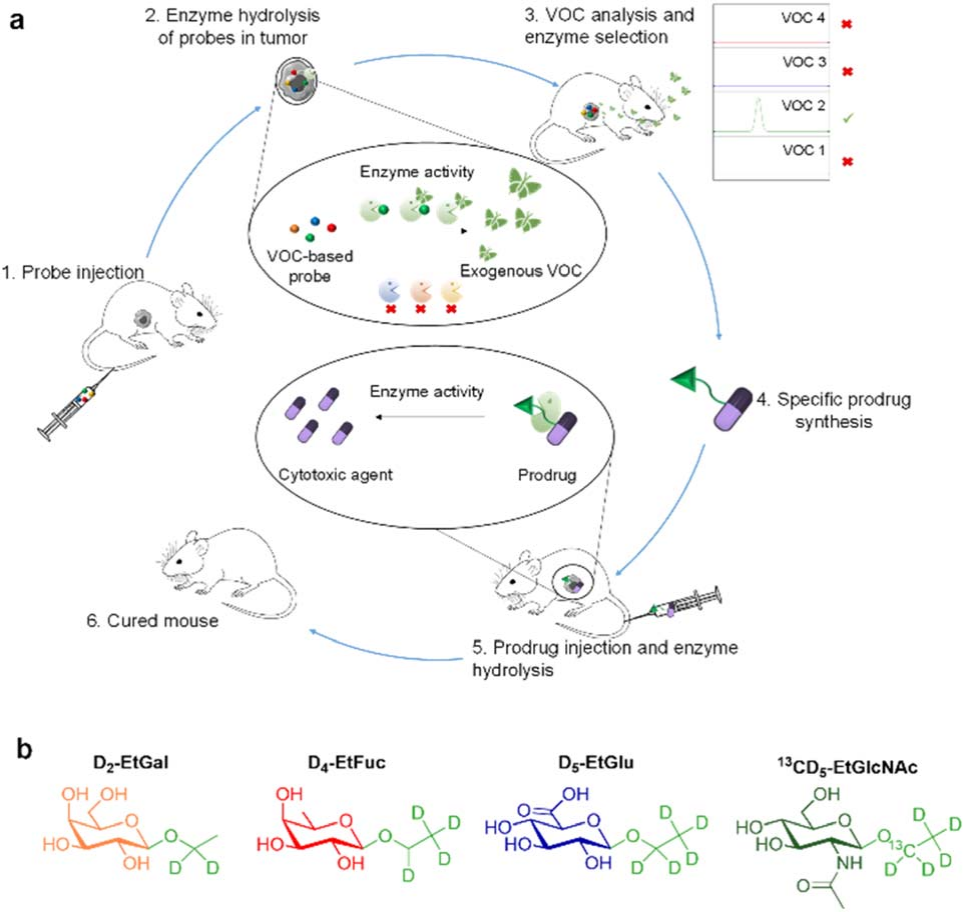
Volatolomics-based modalities for drug discovery. (a) To highlight the dysregulation of glycosidase activity in tumours, a cocktail of VOC-based probes (D_2_-EtGal, D_4_-EtFuc, D_5_-EtGlu and ^13^CD_5_-EtGlcNAc) is administered to tumour-bearing mice (1). The probes can be hydrolysed selectively in the tumour microenvironment by the corresponding glycosidases to release D_2_, D_4_, D_5_ or ^13^CD_5_-ethanol isotopes (2). When released in tumours, labelled VOCs pass in the blood stream and are exhaled in the breath (3). Analysis of VOC signals permits the identification of the most active enzyme in the tumour microenvironment. The corresponding enzyme-responsive drug delivery system is synthesized (4), injected in mice (5) and evaluated for its anticancer efficacy in mice (6). (b) Chemical structure of D_2_-EtGal, D_4_-EtFuc, D_5_-EtGlu and ^13^CD_5_-EtGlcNAc probes.

In this proof-of-concept, we chose to target glycolytic activity of tumours since glycosidase dysregulation constitutes a hallmark of many cancers.^22–26^ β-Galactosidase (β-Gal), α-l-fucosidase (α-Fuc), β-glucuronidase (β-Glu) and *N*-acetyl-β-d-glucosaminidase (β-GlcNAc) were selected as the target enzymes since their overexpression has been reported in several solid tumours.^27–34^ Thus, the probe cocktail was composed of four ethyl glycosides *i.e.* D_2_-ethyl-β-d-galactopyranoside (D_2_-EtGal), D_4_-ethyl-α-l-fucopyranoside, (D_4_-EtFuc), D_5_-ethyl-β-d-glucuronide (D_5_-EtGlu) and ^13^CD_5_-ethyl-*N*-acetyl-β-d-glucosamine (^13^CD_5_-EtGlcNAc), designed to release D_2_-, D_4_-, D_5_- and ^13^CD_5_-ethanol isotopes, respectively, in the presence of the corresponding glycosidase.

After cocktail administration (Fig. 1, step 1), VOC-based probes can be converted into labelled ethanol exclusively if the corresponding glycosidase is overexpressed in the tumour mass (step 2). The volatile tracers can diffuse in the blood stream and be monitored in an animal's breath as reporters of the glycolytic activity prevailing in the tumour (step 3). This screening phase permitted to identify β-GlcNAc as a target for the development of a tumour activated therapy. On this basis, we designed and evaluated (steps 4 and 5) the first β-GlcNAc prodrug of the potent monomethyl auristatin E (MMAE) that demonstrated a remarkable therapeutic efficacy on orthotopic triple-negative mammary tumour in mice (step 6).

To ensure the selective detection of D_2_-, D_4_-, D_5_- and ^13^CD_5_-ethanol molecules, we first developed an analytical strategy involving the GC-MRM-MS methodology (see the ESI† for more details).

We validated its performance by drawing calibration curves for each labelled ethanol, in the absence and presence of the three other isotopes (Fig. 2). The superposition of both curves indicates that the isotope signals did not interfere with each other.

**Fig. 2:**
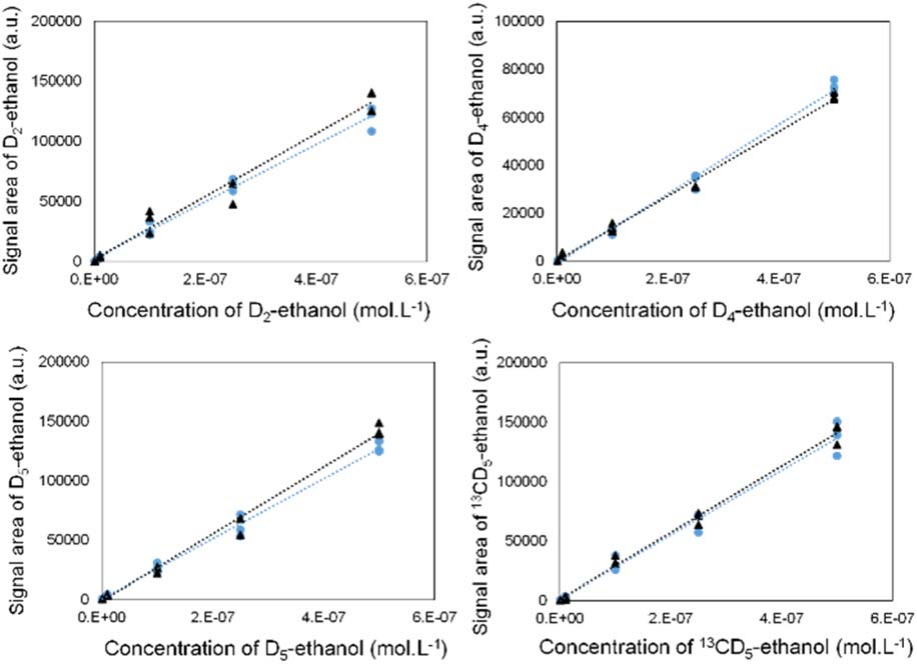
Calibration curves for solutions of D_2_-ethanol, D_4_-ethanol, D_5_-ethanol and ^13^CD_5_-ethanol in the absence (black regression lines) or in the presence of the three other isotopes (blue regression lines).

Since β-Gal, α-Fuc, β-Glu and β-GlcNAc are lysosomal enzymes widely distributed in healthy tissues, we next evaluated the dose of probes that has to be injected in mice for avoiding unselective activation. Indeed, at too high doses, probes can penetrate across cell membranes and then be hydrolysed by intracellular glycosidases in non-target tissues. Thus, we conducted preliminary experiments in healthy mice to determine the lowest detectable signal (LDS) for each VOC-based probe. For this purpose, we administered increasing probe concentrations to the animals that were then placed for over 1 h 30 in airtight cages equipped with an SPME fibre (Fig. 3a).

**Fig. 3:**
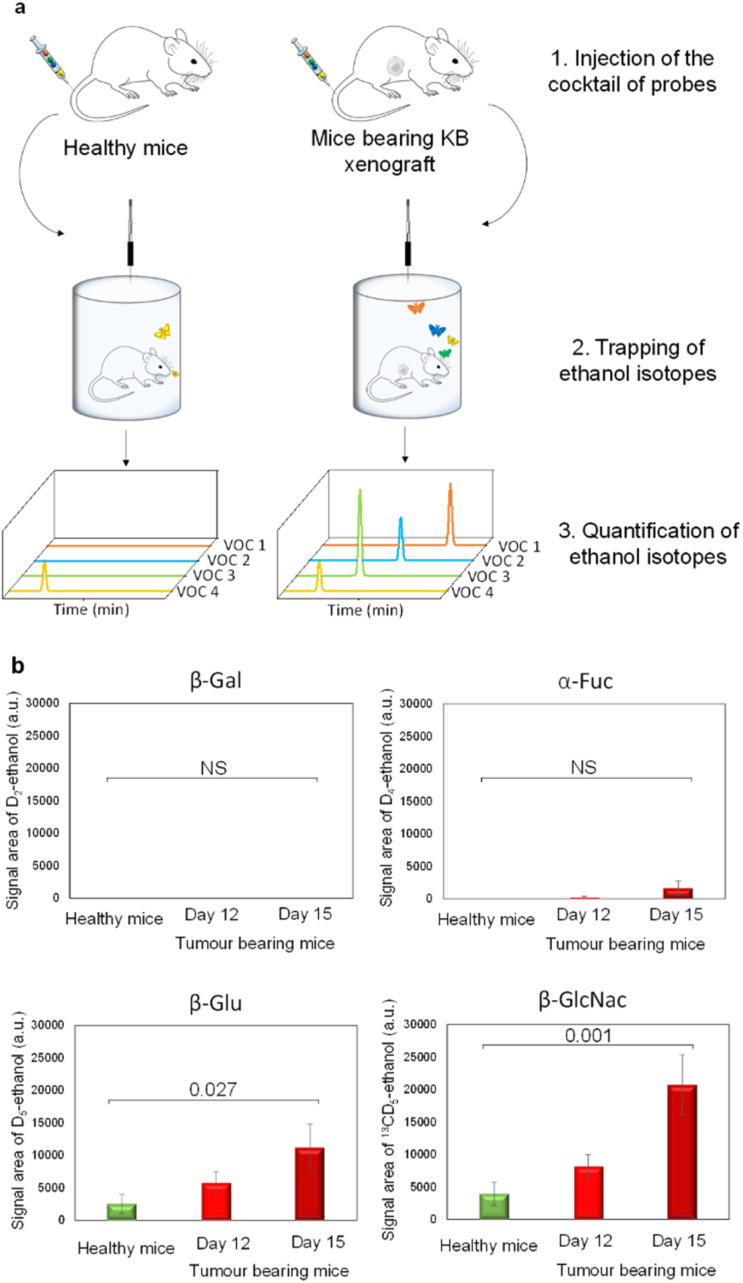
(a) *In vivo* protocol to detect the presence of exoglycosidases in the tumour microenvironment. (b) Amount of D_2_-ethanol, D_4_-ethanol, D_5_-ethanol and ^13^CD_5_-ethanol exhaled by athymic nude healthy mice (green bars; *n* = 6); amount of D_2_-ethanol, D_4_-ethanol, D_5_-ethanol and ^13^CD_5_-ethanol exhaled by mice with a KB subcutaneous xenograft 12 days post tumour implantation (red bars, *n* = 6); amount of D_2_-ethanol, D_4_-ethanol, D_5_-ethanol and ^13^CD_5_-ethanol exhaled by mice with a KB subcutaneous xenograft 15 days post tumour implantation (dark red bars, *n* = 6). For each isotope, significant differences in ethanol isotope amounts were investigated by ANOVA (confidence interval of 95%). Numbers above square brackets correspond to *p*-values.

During the last 30 minutes, exhaled D_2_-, D_4_-, D_5_- and ^13^CD_5_-ethanol isotopes were trapped and subsequently analysed by GC-MRM-MS. Under these conditions, injection of 1, 100, 10 and 10 μg kg^−1^ doses of D_2_-EtGal, D_4_-EtFuc, D_5_-EtGlu and ^13^CD_5_-EtGlcNAc respectively, produced the LDS for the corresponding volatile tracers (see the ESI†). Thus, these probe concentrations were selected for the selective detection of glycosidases overexpressed in the tumour extracellular matrix.

Thereafter, our experimental protocol was applied to highlight the potential upregulation of glycosidases in malignant tissues. The cocktail of probes was administered to athymic nude mice bearing subcutaneous human KB xenografts (cervical cancer cell line) on days 12 and 15 post-tumour implantation. A control experiment was also carried out with healthy animals. On days 12 and 15, the amounts of D_5_- and ^13^CD_5_-ethanol were significantly higher in the breath of cancer animals compared to those emitted by their healthy counterparts (Fig. 3b). In contrast, the concentrations of D_2_- and D_4_-ethanol exhaled by tumour-bearing mice remained in the background noise. These results indicate that both β-Glu and β-GlcNAc exhibit increased catalytic activities in tumours while β-Gal and α-Fuc are not upregulated. Although the overexpression of β-Glu in solid tumours has been widely reported in the literature,^35,36^ the presence of elevated concentrations of β-GlcNAc remains weakly documented so far.^37,38^ These outcomes emphasize the potential of induced-volatolomics modalities to identify novel enzyme targets for drug discovery. Interestingly, between days 12 and 15, enzymatic activities of β-Glu and β-GlcNAc increased concomitantly with tumour growth (1.9- and 2.5-fold, respectively). Under such circumstances, the VOC-based probe cocktail seems to be a useful tool for monitoring disease progression in real time.^21^

We next investigated our probe cocktail *ex vivo* on tumour biopsies. The aim of this trial was to compare simultaneously the catalytic activity of the four glycosidases in tumours and healthy tissues such as kidneys. In these experiments, we used D_2_-EtGal, D_4_-EtFuc, D_5_-EtGlu and ^13^CD_5_-EtGlcNAc probes at 10^−6^, at 10^−4^, 10^−6^, and 10^−7^ mol L^−1^, respectively, since these concentrations produced the LDS for ethanol isotopes when used on healthy tissues (see the ESI†).

Thus, MDA-MB-231 human mammary tumours harvested from nude mice with orthotopic xenografts were incubated in acetate buffer (pH 5) in the presence of the cocktail of VOC-based probes (Fig. 4a). Volatile tracers released in the sample headspace were monitored by GC-MRM-MS (Fig. 4b). The same experiment was also conducted with mice kidneys. Excepted for D_2_-ethanol, concentrations of the volatile tracers released in the headspace were higher for tumour than for kidney samples. These results indicated the presence of an elevated level of α-Fuc, β-Glu and β-GlcNAc in MDA-MB-231 xenografts compared to in healthy tissues. In contrast, β-Gal activity in tumours was highly variable and not significantly different than that of the control group. As was the case for KB tumours, β-GlcNAc was identified once again as the most active glycosidase. Indeed, ^13^CD_5_-ethanol was the volatile tracer exhibiting the highest GC-RMM-MS signal, although ^13^CD_5_-EtGlcNAc was the probe incubated at the lowest concentration (10^−7^ mol L^−1^).

**Fig. 4:**
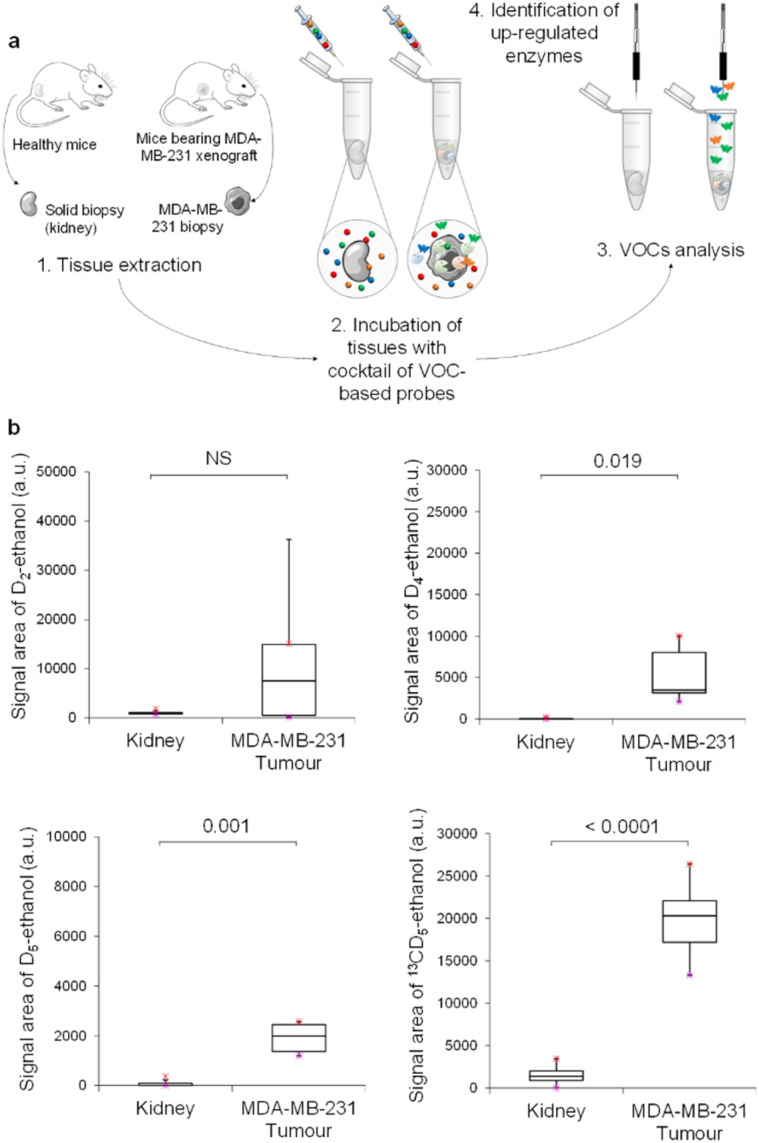
(a) *Ex vivo* protocol for the detection of exoglycosidases on solid biopsies. Healthy kidneys and MDA-MB-231 grafts were incubated in the presence of a cocktail of VOC-based probes (D_2_-EtGal, D_4_-EtFuc, D_5_-EtGlu and ^13^CD_5_-EtGlc). The four ethanol isotopes were trapped during 30 min in the sample headspace and analyzed by GC-MRM-MS. (b) Chemical evidence of β-galactosidase, α-l-fucosidase, β-glucuronidase and *N*-acetyl-β-d-glucosaminidase activities in tissue samples. The results are presented in the form of boxplot representations. Purple and red crosses correspond to the minimum and maximum isotope signals, respectively. Eight kidneys and six MDA-MB-231 xenografts were used in this experiment. Numbers above square brackets correspond to *p*-values.

Overall, these results suggest that the overexpression of β-GlcNAc could be a hallmark of solid tumours. Therefore, this glycosidase appeared to be an attractive target for the development of a novel enzyme-activated cancer therapy. With the aim to explore this therapeutic strategy, we designed the β-GlcNAc-responsive albumin-binding prodrug 1 programmed for the selective delivery of MMAE in the tumour microenvironment^39–41^ (Fig. 5a). Once in the blood stream, 1 is expected to bind selectively to the free thiol of circulating albumin through Michael addition. The resulting bioconjugate will then accumulate in the tumour microenvironment where the glycosidic bond will be cleaved by extracellular β-GlcNAc, hence triggering the release of MMAE *via* the self-immolative mechanism^42–44^ described in Fig. 5a. It is worth mentioning here that glycosidase-responsive albumin-binding prodrugs are more efficient than their non-binding counterparts since they lead to increased drug deposition in the tumor microenvironment combined with a prolonged plasma half-life.^39,45^

**Fig. 5:**
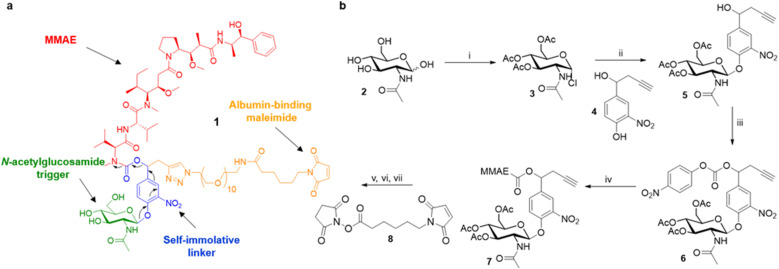
(a) Structure of the β-GlcNAc-responsive albumin-binding prodrug 1 (b) synthesis of the *N*-acetylglucosamide prodrug 1 (i) acetyl chloride (14 eq.), RT, 72 h, 61%; (ii) 4 (1.5 eq.), tetrabutylammonium bromide (1 eq.), CH_2_Cl_2_/NaHCO_3_ 1 M, RT, 5.5 h, 68%; (iii) 4-nitrophenyl chloroformate (2 eq.), pyridine (2.5 eq.), CH_2_Cl_2_, 0 °C to RT, 3 h, 72%; (iv) MMAE (1 eq.), HOBt (1 eq.), pyridine/DMF, RT, 72 h, 93%; (v) *O*-(2-Aminoethyl)-*O*′-(2-azidoethyl)nonaethylene glycol (1.1 eq.), Cu(MeCN)_4_PF_6_ (1 eq.), CH_2_Cl_2_, RT, 3 h; (vi) MeONa (0.15 eq.), MeOH, RT, 5 h; (vii) 8 (1.1 eq.), Et_3_N (3 eq.), DMSO, RT, 1 h, 24% over 3 steps.

The tumour-activated prodrug 1 was accessible from the commercially available β-*N*-acetylglucosamide 2 (Fig. 5b). First, this latter one was converted into the glycoside donor 3 (61%) that was coupled subsequently with the nitrophenol 4 (ref. 46 and 47) to afford compound 5 in 68% yield. Treatment of the benzyl alcohol 5 with 4-nitrophenyl chloroformate led to the activated carbonate 6 (72%). MMAE was then introduced *via* nucleophilic substitution in the presence of hydroxybenzo-triazole to give the carbamate 7 (93%). The three last steps of the synthesis were undertaken without intermediate purification. Thus, alkyne 7 reacted with *O*-(2-aminoethyl)-*O*-(2-azidoethyl)-nonaethylene glycol and Cu(CH_3_CN)_4_PF_6_ to form the corresponding triazole through copper(i)-catalysed azide–alkyne 1,3-cycloaddition. Deprotection of the glycoside followed by the introduction of the maleimide moiety using the *N*-hydroxysuccinimide ester 8 finally led to the drug delivery system 1 (24% over 3 steps).

As enzymatic hydrolysis of the β-*N*-acetylglucosamide trigger is the key step in the process of drug release, we first verified that the carbohydrate moiety was still accessible to β-GlcNAc once bound to albumin. For this purpose, prodrug 1 was incubated with human serum albumin (HSA) at 37 °C in order to form the corresponding bioconjugate through Michael addition. Under these conditions, nearly 90% of 1 was converted in two hours as a result of its rapid binding with the protein (Fig. 6a). The formation of the albumin conjugate was confirmed by trypsin digestion followed by HPLC/HRMS analysis (see the ESI†). When β-GlcNAc was incubated in the medium, the full release of MMAE was achieved in three hours indicating that the β-*N*-acetylglucosamide was a substrate of the enzyme for the activating enzyme even with the proximity of the bulky albumin (Fig. 6b). In contrast, the release of MMAE was not observed after 22 hours of incubation in the absence of β-GlcNAc (see the ESI†). Thus, these results highlighted that the prodrug 1 possessed the capacity both to bind covalently to HSA and release MMAE following enzymatic activation.

**Fig. 6:**
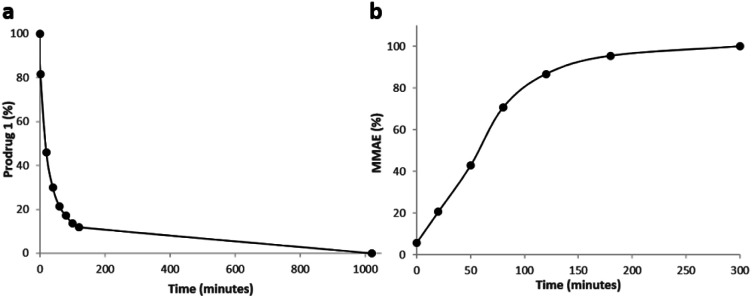
(a) Disappearance of 1 over time when placed in the presence of HSA at 37 °C. (b) Kinetics of MMAE release from 1 linked to HSA in the presence of β-GlcNAc (0.227 U mL^−1^).

We then examined the antiproliferative activity of *N*-acetylglucosamide 1 against human KB and MDA-MB-231 tumour cell lines. As shown in Fig. 7a, the prodrug 1 exhibited a similar cytotoxicity to free MMAE, with IC_50_ values in the low nanomolar range. This result indicated that 1 was activated by β-GlcNAc produced by tumour cells,^38^ leading to the release of the drug. Since the co-incubation of the prodrug with β-GlcNAc did not induce a supplementary cytotoxicity, it appeared that the level of the glycosidase generated by tumour cells in the culture medium was sufficiently elevated for the full activation of 1. In order to confirm that the cytotoxicity of the β-*N*-acetylglucosamide 1 was due to its enzymatic activation in the culture medium, we investigated the antiproliferative activities of the galactoside^48^ and glucuronide^39^ analogues against MDA-MB-231 tumor cells (see the ESI†). In contrast to 1, when these prodrugs were incubated in the absence of the corresponding enzyme (β-Gal or β-Glu), they appeared far less toxic than MMAE. On the other hand, addition of the activating enzyme in the culture medium triggered a strong antiproliferative activity due to the enzyme-catalysed release of the drug. As β-galactoside, β-glucuronide and β-*N*-acetylglucosamide prodrugs differ exclusively by the nature of their glycoside triggers, these results showed that the cytotoxicity observed on MDA-MB-231 tumor cells is due to the β-GlcNAc-mediated activation of 1.

**Fig. 7:**
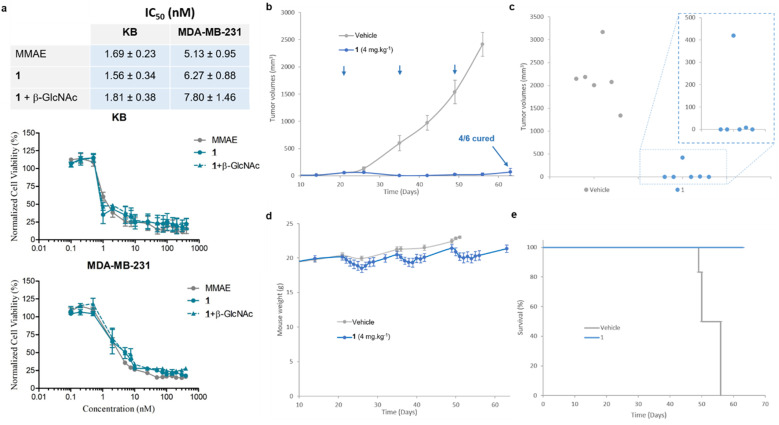
(a) Antiproliferative activities and IC_50_ of MMAE and 1 with or without β-GlcNAc after 3 days of treatment. Each point shows mean ± s.e.m. from 3 independent experiments in triplicate. (b) MDA-MB-231 tumour growth inhibition under therapy with a vehicle and 1. Each point shows mean ± s.e.m. from 6 tumour volumes. (c) Post-euthanasia tumour volumes of mice bearing MDA-MB-231 xenografts treated with a vehicle and 1. (d) Mean body weights of each group of mice bearing MDA-MB-231 xenografts. Each point shows mean ± s.e.m. from 6 mice. (e) Survival study comparing mice with MDA-MB-231 tumours treated with 1 or untreated.

We next pursued our study by assessing the antitumour activity of the β-GlcNAc-sensitive prodrug 1 in Balb/c athymic mice bearing orthotopic MDA-MB-231 triple-negative mammary tumours (Fig. 7). The animals received i.v. injections of 1 at 4 mg kg^−1^ once every two weeks. Following this protocol, a remarkable reduction of tumour volume was observed in all treated animals (Fig. 7b). At the end of the experiment (day 63), 66% of the mice treated with 1 exhibited complete remission (Fig. 7c). In contrast, a previous study showed that the free MMAE produced only a poor anticancer activity, even when administered at its maximal tolerated dose.^39^ The successive i.v. administrations of 1 (1.54 mg per kg per injection MMAE equivalents) were well tolerated without major body weight loss (Fig. 6d) or detectable side effects. These outcomes demonstrated that the derivatization of MMAE in the form of the β-*N*-acetylglucosamide 1 significantly reduced its toxicity *in vivo*, allowing the administration of at least 3-fold the maximal tolerate dose (MTD) of the drug.^39^ Furthermore, the therapeutic efficacy obtained with 1 confirmed the selective and efficient enzyme-mediated release of MMAE in the tumour mass. Thus, this study showed that the targeting of tumour-associated β-GlcNAc by means of the enzyme-responsive albumin-binding prodrug is a promising therapeutic strategy for the treatment of triple-negative breast cancer (TNBC), a clinically aggressive disease for which current drugs are ineffective.

## Conclusions

In this study, we demonstrated that a cocktail of VOC-based probes allowed the screening of several tumour-associated enzyme activities in both living mice and tumour biopsies. This induced-volatolomics modality led to the identification of an enzyme target, β-GlcNAc, which was exploited for the selective delivery of an anticancer drug that produced an impressive anticancer activity in mice bearing TNBC xenografts. Hence, our work highlights the potential of induced-volatolomics for the discovery of tumour-associated enzyme targets and the development of novel therapeutic strategies. Numerous VOC-based probe cocktails can be designed for the multimodal exploration of enzymatic dysregulation related to various diseases. Thus, induced-volatolomics modalities could become powerful tools to accelerate the process of drug discovery in the near future.

## Ethical statement

Experimental procedures involving animals were conducted according to protocol no. 2017053011069010 approved by the national ethical committee and carried out in accordance with the guidelines of the French Agriculture and Forestry Ministry (decree 2013-118) and the European Communities Council Directive (2010/63/UE).

## Data availability

All relevant data supporting this article have been included in the main text and the ESI.†

## Author contributions

P. P. and S. P. supervised the whole study. P. P., C. G. and M. T. supervised the induced-volatolomics approach and the development of analytical methods. P. P. and J. C. designed *in vivo* studies to test the efficiency of the cocktail of probes for highlighting highly active enzymes in tumours. J. L., J. C., F. D. and E. B. performed the *in vitro* and *in vivo* experiments using VOC-based probes. R. E. completed the VOC-based probe sensitivity study. J. C. and E. B. performed cell viability assays. S. P., I. O., B. R., R. C. and W. T. designed and synthesized the anticancer agent and VOC-based probes. R. C. and R. E. performed biochemical assays to certify the formation of the albumin–prodrug conjugate. J. S. and A. L. P. designed and supervised the experiments in tumour bearing mice treated with the enzyme-responsive prodrug. J. L., E. B. and R. C. collected all the data and reconstructed the figures. J. L., E. B. and R. C. wrote the methods section. P. P. and S. P. wrote the paper with the contribution of all authors.

## Conflicts of interest

There are no conflicts to declare.

## Supplementary Material

SC-014-D2SC06797H-s001
