# Supplementary material for: Induced-volatolomics for the design of tumour activated therapy
Source: Chem Sci. 2023 Apr 11;14(18):4697–703. doi: 10.1039/d2sc06797h (PMC10171039; doi:10.1039/d2sc06797h)
Supplement: SC-014-D2SC06797H-s001 [file SC-014-D2SC06797H-s001.pdf]

Electronic Supplementary Material (ESI) for Chemical Science.
This journal is © The Royal Society of Chemistry 2023

## SUPPORTING INFORMATION

# Induced-volatolomics for the design of tumour activated therapy

Rémi Châtre[a], Estelle Blochouse[a], Rony Eid[a], Fabiola Djago[a], Justin Lange[a], Mehrad Tarighi[a], Brigitte Renoux[a], Julien Sobilo[b], Alain Le Pape[b], Jonathan Clarhaut[a, c], Claude Geffroy[a], Isabelle Opalinski[a], Wei Tuo[a], Sébastien Papot*[a, d], and Pauline Poinot*[a]

**Abstract:** *The discovery of tumour-associated markers is of major interest for the development of selective cancer chemotherapy. Within this framework, we introduced the concept of induced-volatolomics enabling to monitor simultaneously the dysregulation of several tumour-associated enzymes in living mice or biopsies. This approach relies on the use of a cocktail of volatile organic compounds (VOCs)-based probes that are activated enzymatically for releasing the corresponding VOCs. Exogenous VOCs can be then detected in the breath of mice or in the headspace above solid biopsies as specific tracers of enzyme activities. Our induced-volatolomics modality highlighted that the up-regulation of N-acetylglucosaminidase was a hallmark of several solid tumours. Having identified this glycosidase as a potential target for cancer therapy, we designed an enzyme-responsive albumin-binding prodrug of the potent monomethyl auristatin E programmed for the selective release of the drug in the tumour microenvironment. This tumour activated therapy produced a remarkable therapeutic efficacy on orthotopic triple-negative mammary xenografts in mice, leading to the disappearance of tumours in 66 % of treated animals. Thus, this study shows the potential of induced-volatolomics for the exploration of biological processes as well as the discovery of novel therapeutic strategies.*

## Table of content

I. Induced volatolomics modality ..... 3
1. Chemicals ..... 3
2. Cell lines ..... 3
3. Animals ..... 3
4. Volatile Organic Compound preconcentration and analysis ..... 3
5. VOC-based probes sensitivity ..... 4
6. *In vivo* experiments ..... 6
7. *In vitro* experiments ..... 6
8. Statistical analysis ..... 7

II. Chemistry Section ..... 7
1. General experimental methods ..... 7
2. Synthesis of prodrug 1 ..... 8
3. Trypsin-digestion experiments ..... 21
4. Stability and enzymatic cleavage ..... 22

III. Biological evaluation of prodrug 1 ..... 23
1. Cell viability ..... 23
2. *In vivo* experiments ..... 24

References ..... 24

# SUPPORTING INFORMATION

## I. Induced volatolomics modality

### 1. Chemicals

RPMI 1640-GlutaMAX and Penicillin/Streptomycin were obtained from Life Technologies (Illkirch, France). Fetal bovine serum and Matrigel® were purchased from Lonza (Verviers, Belgium) and Sigma-Aldrich (Saint-Quentin Fallavier, France), respectively.

$D_2$-Ethyl-β-D-galactoside ($D_2$-EtGal), $D_4$-ethyl-α-D-fucoside ($D_4$-EtFuc), $D_5$-ethyl-β-D-glucuronide ($D_5$-EtGlu) and $^{13}CD_5$-N-acetyl-β-D-glucosamide ($^{13}CD_5$-EtGlc) were synthesized by Seekyo/IC2MP (Poitiers, France) according to the general synthetic procedure previously described in Lange *et al.*[1] Standards of ethanol ($D_2$-ethanol, $D_4$-ethanol, $D_5$-ethanol and $^{13}CD_5$-ethanol) were purchased from Sigma-Aldrich.

### 2. Cell lines

For *in vivo* assays, Human KB (Cervix cancer, ATCC CCL-17) cell line was purchased from LGC Standards (Molsheim, France). For *in vitro* assays, MDA-MB-231 (breast adenocarcinoma, ATCC HTB-26) cell line was purchased from Sigma-Aldrich. Cells were maintained in RPMI 1640-GlutaMAX supplemented by 10% fetal bovine serum, and 100 $u.ml^{-1}$ penicillin/streptomycin (Life Technologies) and implanted to mice at early passages (<5).

### 3. Animals

Experimental procedures involving animals were conducted according to the protocol n° 2017053011069010 approved by the national ethical committee and carried out in accordance with the guidelines of the French Agriculture and Forestry Ministry (decree 2013-118) and the European Communities Council Directive (2010/63/UE).

Athymic nude-*Foxn1^nu^* mice (Envigo, Gannat, France) were used. Seven-weeks old mice were anesthetized by inhalation of 2% vaporized isoflurane. A quantity of $1.10^6$ cells in a phosphate-buffered saline 1X – 50% Matrigel® solution were transplanted subcutaneously into their dorsal flank region (day 0).

Tumor sizes were measured two times a week in two dimensions using digital caliper. Individual tumor volumes were calculated by the formula[2]5: Volume ($mm^3$) = (X) × ($Y^2$) / 2, where X was the largest diameter (mm) and Y the smallest (mm). Mice weights were also monitored two times a week. In accordance with the guidelines of the European Communities Council Directive (2010/63/UE), mice were euthanized when tumor exceeded a volume of 2 $cm^3$, when necrosis appeared or at the end of the experiment.

For *in vivo* assays, 6 Athymic nude mice were transplanted with human KB tumor grafts and 14 were used as control. For *in vitro* assays, 6 Athymic nude mice were transplanted with human MDA-MB-231 tumor grafts and 8 healthy mice were used as control. The mice were sacrificed 35 to 56 days after the tumor transplantation ant the tumor volumes varies between 577 and 2452$mm^3$. Tumors and kidneys were surgically resected and conserved at -80 °C.

### 4. Volatile Organic Compound preconcentration and analysis

VOCs were trapped on a 75 µm carboxen/polydimethylsiloxane (CAR/PDMS) SPME fibre (Supelco, Bellffone, USA). VOCs were analyzed with a GC-MS/MS system that consisted of a Trace 1300 Thermo Scientific gas chromatograph coupled with a TSQ 9000 triple quadrupole mass spectrometer (Thermo Fisher Scientific, Waltham, MA, USA) operated in multiple reaction monitoring (MRM, GC-MRM MS).

After SPME fiber desorption, VOCs were separated on a non-polar capillary column DB-624 60 m x 250 µm x 1,4 µm (Agilent). The injector temperature was set at 260°C in splitless mode. Septum purge flow and gas saver flow were set to 5 $mL.min^{-1}$ and 20 $mL.min^{-1}$ respectively. SPME desorption was performed with helium as the carrier gas with a flow of 1.2 $mL.min^{-1}$. An isotherm program was used to separate VOCs on the column. Oven temperature was set at 90 °C during 10 min.

Multiple reaction monitoring (MRM) transitions and collision energies for the four ethanol isotopes are summarized in Table S1.

| Compound | Precursor | Fragment | Collision energy (eV) |
|---|---|---|---|
| $D_2$-ethanol | 48.1 | 33.1 | 5 |
| $D_4$-ethanol | 50.1 | 32.1 | 10 |
| $D_5$-ethanol | 51.1 | 33.1 | 10 |
| $^{13}$CD5-ethanol | 52.1 | 34.1 | 6 |

**Table S1.** MRM transitions of labelled ethanol molecules.

To assess the selectivity of this MRM MS method, a five-points calibration curve ranging from $1.10^{-9}$ to $5.10^{-7}$ mol.L$^{-1}$ was constructed for each of the four ethanol isotopes ($D_2$, $D_4$, $D_5$, $^{13}CD_5$) (three replicates per calibration point). Ethanol molecules were trapped during 30 min with a SPME fiber after 2 h 00 incubation at 37°C. The amount of labelled-ethanol trapped on the SPME fiber was analyzed with the GC-MRM MS method. Two calibration curves were constructed for each labelled ethanol, in the absence and in the presence of the three other isotopes (Figure 2).

Every day, trapping efficiency of SPME fibers was verified using a 100 µL solution at $10^{-7}$ mol.L$^{-1}$ of $D_2$-ethanol, $D_4$-ethanol, $D_5$-ethanol, $^{13}CD_5$-ethanol in ultra-pure water. Headspace-SPME trapping was performed during 30 min at 37 °C. Ethanol isotopes were analyzed with the GC-MRM MS method described above.

## 5. VOC-based probes sensitivity

$D_5$-EtGlu was used as model probe. Its enzymatic detection sensitivity was assessed on commercial β-Glu (Sigma Aldrich, Mannheim, Germany). Eight-points calibration curve ranging from 2.5 to 250 U.L$^{-1}$ in β-Glu activity was constructed (two replicates per calibration point). The enzyme was incubated in 300 µL of 0.1 M acetate buffer (pH 5) in the presence of $1.10^{-6}$ M of $D_5$-EtGlu for 30 min (incubation temperature 37 °C). $D_5$-Ethanol was trapped on a SPME fiber during these 30 min and analyzed with the GC-MRM MS as described above. From this calibration curve, we determined an experimental detection limit (LOD) for this commercial β-Glu of 13 U.L$^{-1}$ (Figure S1).

In parallel, eight-points calibration curves were constructed and LODs assessed for two reference probes, i.e. p-nitrophenyl-β-D-glucuronide (Biosynth, Bratislava, Slovakia) and 4-methylumbelliferyl-β-D-glucuronide (Sigma Aldrich, St. Louis, Switzerland), usually employed for β-Glu activity detection.

Briefly, based on the protocol described by Zhang et al.[3], β-Glu (0.2 mL) was incubated in the presence of 0.4 mL of 2 mM p-nitrophenyl-β-D-glucuronide diluted in 0.1 M phosphate buffer (pH 5). The vial was completed with 0.4 mL of 0.1 M acetate buffer (pH 5). The reaction mixture was incubated at 37 °C for 1 h. The reaction was stopped by the addition of 1 mL of 0.5 M NaOH, and the p-nitrophenol (pNP) deconjugated from p-nitrophenyl-β-D-glucuronide (pNPG) was quantified spectrophotometrically at a wavelength of 400 nm. With this method, we determined a LOD of 14 U.L$^{-1}$ for β-Glu (Figure S1).

Following the protocol of Beratis et al.[4], β-Glu (12.5 µL) was incubated in the presence of 4 mM of 4-methylumbelliferyl-β-D-glucuronide in 112.5 µL of 0.1 M acetate buffer (pH 5). The reaction mixture was incubated at 37 °C for 1 h. The reaction was stopped by the addition of 125 µL of cold methanol. The mixture was vortexed and kept at -20 °C for 30 min, followed by a centrifugation at 15000 rpm for 15 min at 4 °C. The resulting supernatant was gently transferred to a new vial and the 4-methylumbelliferon (4-MU) deconjugated from 4-methylumbelliferyl-β-D-glucuronide (4-MUG) was quantified by LC-MS/MS. 4-Methylumbelliferon was analyzed on an Acclaim 120 C18 column (250 mm × 4.6 mm, 5 µm, Thermo Scientific, Europe). Elution was performed at a constant flow of 0.5 mL.min$^{-1}$ at 30 °C. Acetonitrile with 0.1 % formic acid was used as mobile phase A and water with 0.1 % formic acid as mobile phase B. The gradient started with 30 % of A at 3 min and reached 90 % of A in 10 min, then constant for 1 min. A second gradient was used to reach 100 % of A in 4 min, and then constant for 3 min. The column was then reconditioned for 4 min with 30 % of A. TQ MS capillary voltage was set at 4 kV, the interface temperature was held at 300 °C and heat block temperature at 400 °C. The desolvation line temperature was held at 526 °C. Nebulizing gas flow, heating gas flow and drying gas flow were set at 3 L.min$^{-1}$, 10 L.min$^{-1}$ and 10 L.min$^{-1}$ respectively. Multiple reaction monitoring (MRM) transitions were 175.1>119.0 and 175.1>133.0, with dwell times of 100ms both, and 25 eV and 23 eV collision energies respectively. We obtained a LOD of 140 U.L$^{-1}$ for β-Glu (Figure S1).

Therefore, the β-Glu-responsive VOC-based probe can detect up to 10-fold lower concentration of enzymes compared to standard probes.

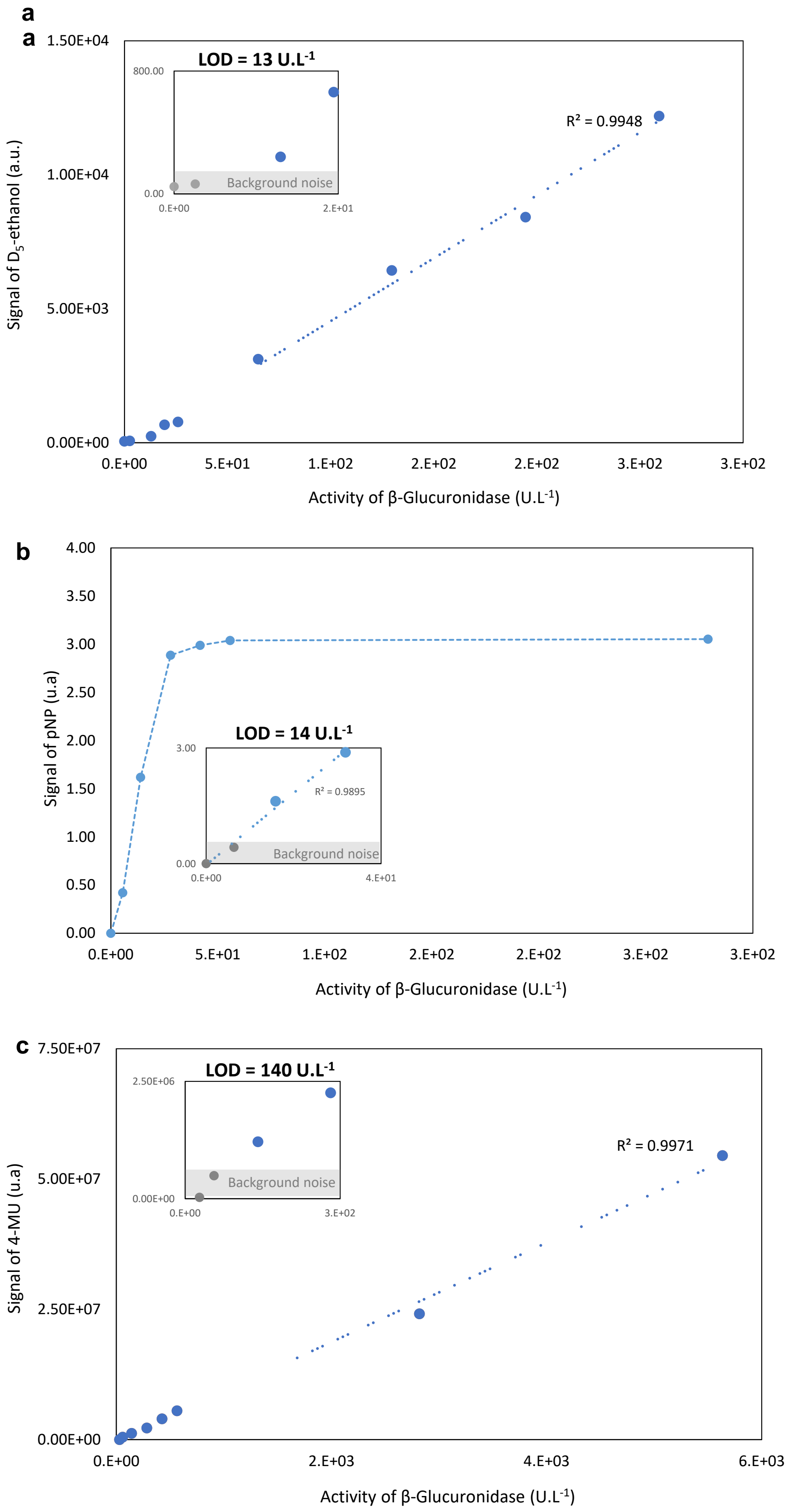


**Figure S1**. Calibration curves for β-Glu activity detection with $D_5$-EtGlu (a), p-nitrophenyl-β-D-glucuronide (b) and 4-methylumbelliferyl-β-D-glucuronide (c). Experimental limits of detection (LODs) are indicated in the graphs.

## 6. *In vivo* experiments

To determine the optimal dose of VOC-based probes to be injected to mice, increasing concentrations of probes were administered in Athymic nude mice. Before injection, a blank experiment was performed by placing the mice in the airtight cage (130 mm x 130 mm x 200 mm) for 10 min. During this duration, a SPME fibre was inserted into the cage headspace (HS) to estimate VOCs that can interfere with the detection of ethanol isotopes in the atmosphere. Once this blank experimentation done, mice were injected with the cocktail of probes at different concentration. After 1 h00, VOCs released by mice were trapped on SPME fibre during 30 min until 1 h 30. Each dose was injected to two mice. Due to the presence in the atmosphere of an interfering VOC that responded to the same transitions as $D_2$-ethanol, signals of this isotope were subtracted to the background noise. A dose of 1 µg.kg$^{-1}$, 100 µg.kg$^{-1}$, 10 µg.kg$^{-1}$ and 10 µg.kg$^{-1}$ of $D_2$-EtGal, $D_4$-EtFuc, $D_5$-EtGlu and $^{13}CD_5$-EtGlc were shown to provide the minimum quantifiable signal for each labelled ethanol (Figure S2).

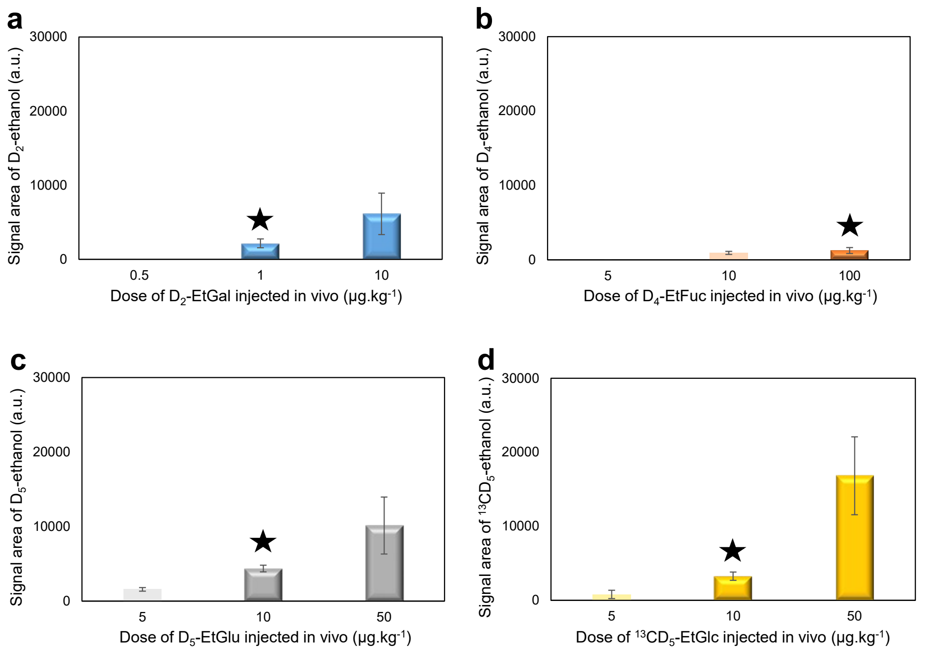


**Figure S2.** Determination of VOC-based probes to be injected for *in vivo* experiments. Each dose was injected to two mice. Selected doses are fitted with a black star. a. Injection of $D_2$-EtGal. b. Injection of $D_4$-EtFuc. c. Injection of $D_5$-EtGlu. d. Injection of $^{13}CD_5$-EtGlc.

For *in vivo* assays, VOC-based probes were injected at optimal concentrations to six athymic nude mice bearing human KB tumor grafts and six healthy athymic nude mice. Before injection, mice were placed inside an airtight cage (130 mm x 130 mm x 200 mm) for 10 min. During this duration, a SPME fibre was inserted into the cage headspace (HS) to estimate VOCs that can interfere with the detection of ethanol isotopes in the atmosphere. Once this blank experimentation done, mice were injected with the cocktail of probes at different concentration. After 1 h00, VOCs released by mice were trapped on SPME fibre during 30 min until 1 h 30. $D_2$-Ethanol signals were subtracted to the background noise due to the presence of an interfering VOC in the atmosphere. Ethanol isotopes were measured in mice breath 12 days post tumor implantation to avoid false- either positive or negative results associated with activity measurement in non-established tumors. Since KB xenografts grow relatively quickly, a three days delay between the first and the second injection of the cocktail is sufficient to highlight an evolution of glycosidases activity.

## 7. *In vitro* experiments

Six MDA-MB tumours and eight kidneys from wild-type Athymic nude mice were placed in a 2 mL clear glass vial. A volume of 270 µL of acetate buffer (pH 5) was added in the vial (incubation temperature 37 °C). A blank experiment was performed by exposing a SPME fibre during 30 min in sample headspace. Trapped VOCs were analysed with the GC-MRM MS method as described above. These blank experiments revealed a low contamination of a few tissues with an interfering VOC answering to the same transitions as $D_2$-ethanol. Thus, when necessary, $D_2$-ethanol signals were subtracted to the background noise.

Thereafter, a volume of 30 µL of the cocktail of $D_2$-EtGal, $D_4$-EtFuc, $D_5$-EtGlu and $^{13}CD_5$-EtGlc probes respectively at $1.10^{-6}$, $1.10^{-4}$, $1.10^{-6}$, $1.10^{-7}$ mol.L$^{-1}$ in acetate buffer was added into the vial. These concentrations were selected from preliminary experiments during which healthy tissues (i.e. kidneys, n=2 per tested concentration) were incubated 30 min with increasing concentrations of probes. VOCs were trapped on SPME fibre during these 30 min. The concentrations that did not provide any $D_2$-, $D_4$-, $D_5$- and $^{13}CD_5$-ethanol signal were selected to emphasize the activity of enzymes present in the extracellular space (Figure S3).

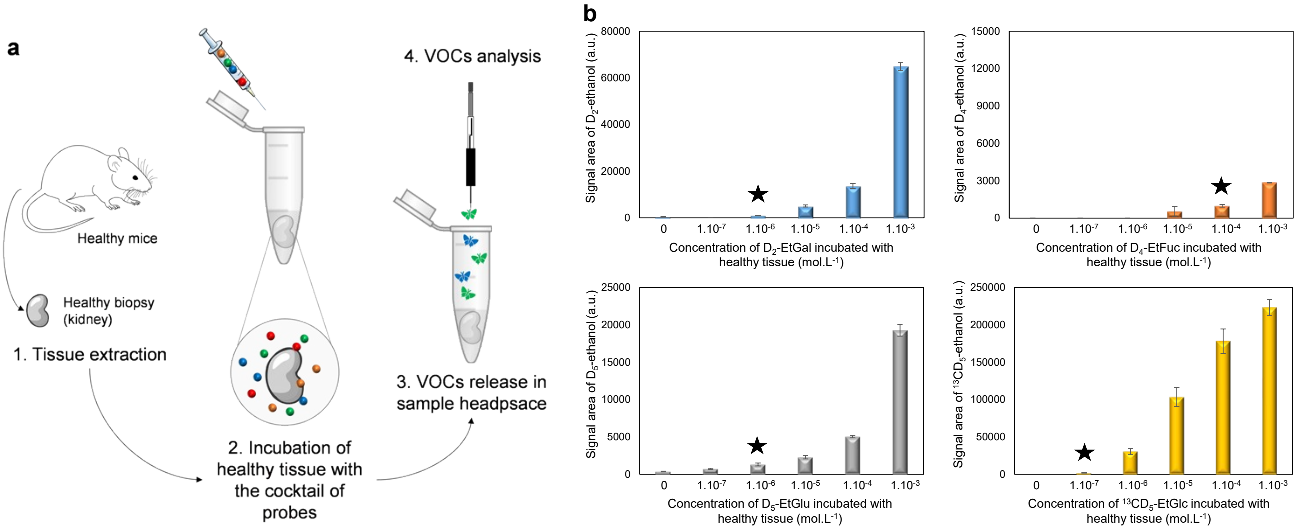


**Figure S3.** Determination of the concentrations of VOC-based probes to incubate with tumour tissues for extracellular enzymes detection. Each concentration was tested on two kidneys. Selected doses are fitted with a black star. a. Injection of $D_2$-EtGal. b. Injection of $D_4$-EtFuc. c. Injection of $D_5$-EtGlu. d. Injection of $^{13}CD_5$-EtGlc.

## 8. Statistical analysis

Data are mean ± s.e.m. All statistical treatments were performed using XLStat 4. Significant differences were determined by one-way ANOVA and Fisher test. A value of p-value $< 0.05$ was considered statistically significant. No statistical analysis was used to predetermine sample sizes. Estimates were made on our previous experience, experimental approaches, availability and feasibility required to obtain statistically significant results. Experimental mice were randomly assigned to each experimental group or control group.

# II. Chemistry Section

## 1. General experimental methods

All reactions were performed under an argon atmosphere. Unless otherwise stated, solvents used were of HPLC quality. Chemicals were of analytical grade from commercial sources and were used without further purification. The reaction progress was monitored either by liquid chromatography, liquid chromatography coupled mass spectroscopy or on precoated silica gel TLC plates MACHEREY-NAGEL ALUGRAM® SIL G/UV254. (0.2 mm silica gel 60). Spots were visualized under 254 nm UV light and/or by dipping the TLC plate into a solution of phosphomolybdic acid (3 g) in ethanol (100 mL) followed by heating with a heat gun.

Automatic chromatographies were performed with a COMBIFLASH® RF 200I TELEDYNE ISCO instrument equipped with UV and ESLD detector and using flash cartridges Interchim® silica 15 or 50 µm for normal phase chromatography and HP C18 RediSep® GOLD 4g or 15,5g for reverse phase chromatography.

$^{1}H$ and $^{13}C$ NMR spectra were respectively recorded at 400 MHz and 100 MHz, on a Bruker 400 Avance III instrument, equipped with an ultrashielded plus magnet and a BBFO 5 mm broadband probe or at 500 MHz and 126 MHz on a Bruker 500 Avance NEO instrument, equipped with an ultrashielded magnet and a Prodigy cryoprobe. Chemical shifts (δ) are reported in parts per million (ppm) from low to high field and referenced to residual solvent peaks. Coupling constants (*J*) are reported in hertz (Hz). Standard abbreviations

indicating multiplicity are used as follows: b = broad, s = singlet, d = doublet, t = triplet, q = quartet, qi = quintet, m = multiplet, dd = doublet of doublets.

High-resolution mass spectra (HRMS) were performed on a LC-QTof MaXis Impact, Bruker.

Analytical LC-MS was performed on a Shimadzu LCMS-2020. A reverse-phase column chromatography MACHEREY-NAGEL NUCLEOSHELL® (150/4.6, RP18, 5 µm) at 40°C was used for chromatographic separation at a flow rate of 1 mL.min$^{-1}$. The column effluent was introduced into the electrospray ionisation source (ESI) of the mass spectrometer. Analyses were performed in positive and negative ion mode. The electrospray voltage was set at 4.5 kV. The capillary and heater temperatures were 250°C and 400°C respectively. The drying gas (nitrogen) and nebulizing gas (nitrogen) flow were set at 15 L.min$^{-1}$ and 1.5 L.min$^{-1}$ respectively. Analysis of data was performed with LabSolutions software. LC-MS experiments were performed using a linear gradient composed of A (0.1% formic acid in water) and B (0.1% formic acid in $CH_3CN$) starting from 20% of B and reaching 100% of B within 15 min.

## 2. Synthesis of prodrug 1

Prodrug **1** was prepared according to the following strategy.

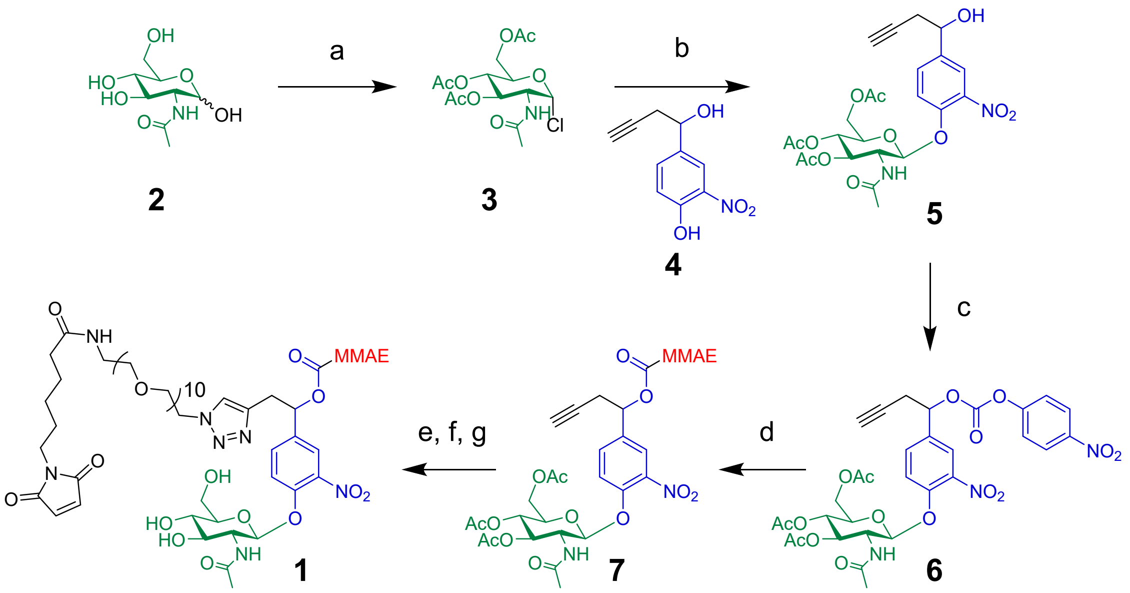


**Scheme S1.** Reagent and conditions: a) Acetyl chloride (14 eq.), RT, 72h, Yield = 61%. b) **4** (1.5 eq.), tetrabutylamonium bromide (1 eq.), $CH_2Cl_2$ / $NaHCO_3$ 1M, RT, 5.5h, Yield = 68%. c) 4-Nitrophenyl chloroformate (2 eq.), pyridine (2.5 eq.), $CH_2Cl_2$, 0°C to RT, 3h, Yield = 72%. d) MMAE (1 eq.), HOBt (1 eq.), pyridine / DMF, RT, 72h, Yield = 93%. e) Azido-PEG10-amine (1.1 eq.), $Cu(MeCN)_4PF_6$ (1 eq.), $CH_2Cl_2$, RT, 3h. f) MeONa (0.15 eq.), MeOH, RT, 5h. g) 6-Maleimidohexanoic acid N-hydroxysuccinimide ester (1.1 eq.), $Et_3N$ (3 eq.), DMSO, RT, 1h, Yield (3 steps) = 24 %.

Synthesis of compound 3

Compound **3** was prepared according to a protocol previously reported in the literature[5].

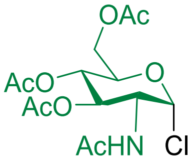


*N*-acetylglucosamine (3.0 g, 13.5 mmol, 1 equiv.) was solubilized in acetyl chloride (13.5 mL, 190 mmol, 14 equiv.) and the solution was stirred for 72 hours at room temperature. After completion, the mixture solution was hydrolyzed by addition of ice water (150 mL), stirred for 5 minutes and $CH_2Cl_2$ (60 mL) was added. The organic layer was separated, washed with saturated $NaHCO_3$ (2 x 60 mL) and brine (60 mL). The combined organic layers were dried over $MgSO_4$, filtrated and concentrated in *vacuo*. The crude residue was purified by chromatography on a silica gel column (PE/AcOEt 50/50 to 0/100 over 20 minutes) to give compound **3** (3.0 g, 61%) as a white solid.

**$R_f$:** 0.37 (PE/AcOEt 40/60)

**$^1$H NMR (400 MHz, $CDCl_3$, 298K):** δ ppm = 6.19 (d, *J* = 3.7 Hz, 1H), 5.77 (d, *J* = 9.0 Hz, 1H), 5.32 (dd, *J* = 14.6, 5.5 Hz, 1H), 5.22 (t, *J* = 9.7 Hz, 1H), 4.53 (ddd, *J* = 10.6, 8.8, 3.8 Hz, 1H), 4.37 - 4,23 (m, 2H), 4.14 (dd, *J* = 13.1, 2.7 Hz, 1H), 2.11 (s, 3H), 2.06 (s, 3H), 2.06 (s, 3H), 1.99 (s, 3H).

Synthesis of compound 5

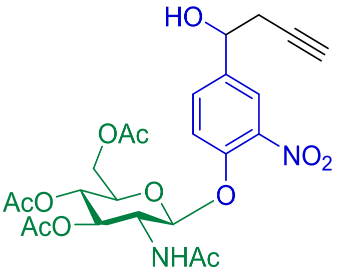


To a solution of the previously reported phenol **4**[5] (169 mg, 0.82 mmol, 1.5 equiv.) solubilized in a mixture of $CH_2Cl_2$ (2 mL) and aqueous $NaHCO_3$ (1M, 1.4 mL) was added tetrabutylammonium iodide (176 mg, 0.54 mmol, 1 equiv.). The mixture was stirred at room temperature for 15 minutes. Then, a solution of compound **3** (200 mg, 0.54 mmol, 1 equiv.) in $CH_2Cl_2$ (1 mL) was added and the mixture was stirred until total consumption of the chlorinated compound **3**. After 5h30, the organic layer was separated, washed with water (5 mL), brine (5 mL), dried over $MgSO_4$, filtrated, and concentrated in *vacuo*. The crude residue was purified by chromatography on a silica gel column (PE/AcOEt 100/0 to 0/100 over 20 minutes) to afford compound **5** (200 mg, 68%) as a white solid.

**$R_f$:** 0.31 ($CH_2Cl_2$/MeOH 95/5)

**$^1$H NMR (500 MHz, $CDCl_3$, 298K):** δ ppm = 7.84 (dd, *J* = 8.1, 2.1 Hz, 1H), 7.59 – 7.50 (m, 1H), 7.35 (d, *J* = 8.6 Hz, 1H), 5.88 (d, *J* = 8.1 Hz, 1H), 5.58 (dd, *J* = 10.4, 9.2 Hz, 1H), 5.49 (dd, *J* = 8.2, 1.4 Hz, 1H), 5.12 (t, *J* = 9.5 Hz, 1H), 4.90 (t, *J* = 6.2 Hz, 1H), 4.27 (dd, *J* = 12.3, 4.6 Hz, 1H), 4.20 (dd, *J* = 12.3, 2.4 Hz, 1H), 3.96 – 3.83 (m, 2H), 2.68 – 2.58 (m, 2H), 2.10 (t, *J* = 2.6 Hz, 1H), 2.09 (s, 3H), 2.06 (s, 3H), 2.05 (s, 3H), 1.98 (s, 3H).

**$^{13}$C NMR (126 MHz, $CDCl_3$, 298K):** δ ppm = 171.3, 170.7, 170.6, 169.6, 148.8, 148.8, 141.5, 141.5, 138.9, 138.9, 131.3, 122.6, 122.6, 121.1, 121.0, 99.7, 79.6, 77.4, 77.2, 76.9, 72.3, 72.1, 71.3, 70.8, 70.8, 68.6, 62.0, 55.4, 29.6, 23.5, 20.9, 20.8, 20.8.

**HRMS (ESI):** $[M+Na]^+$ calcd for $C_{24}H_{28}N_2NaO_{12}$: 559.1534 found 559.1521.

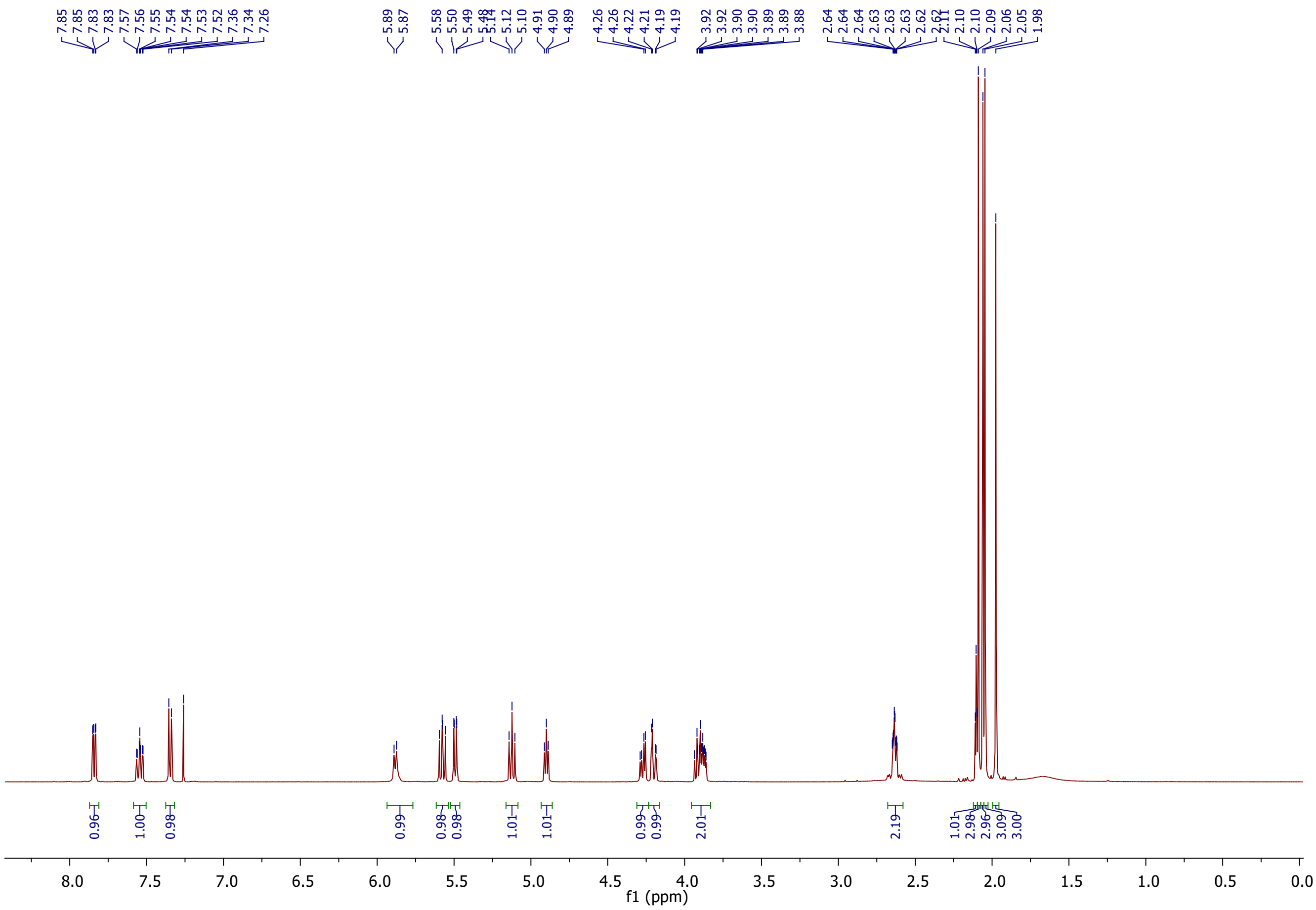

$^{1}$H NMR spectrum (500 MHz, 298 K, $CDCl_3$) of compound **5**

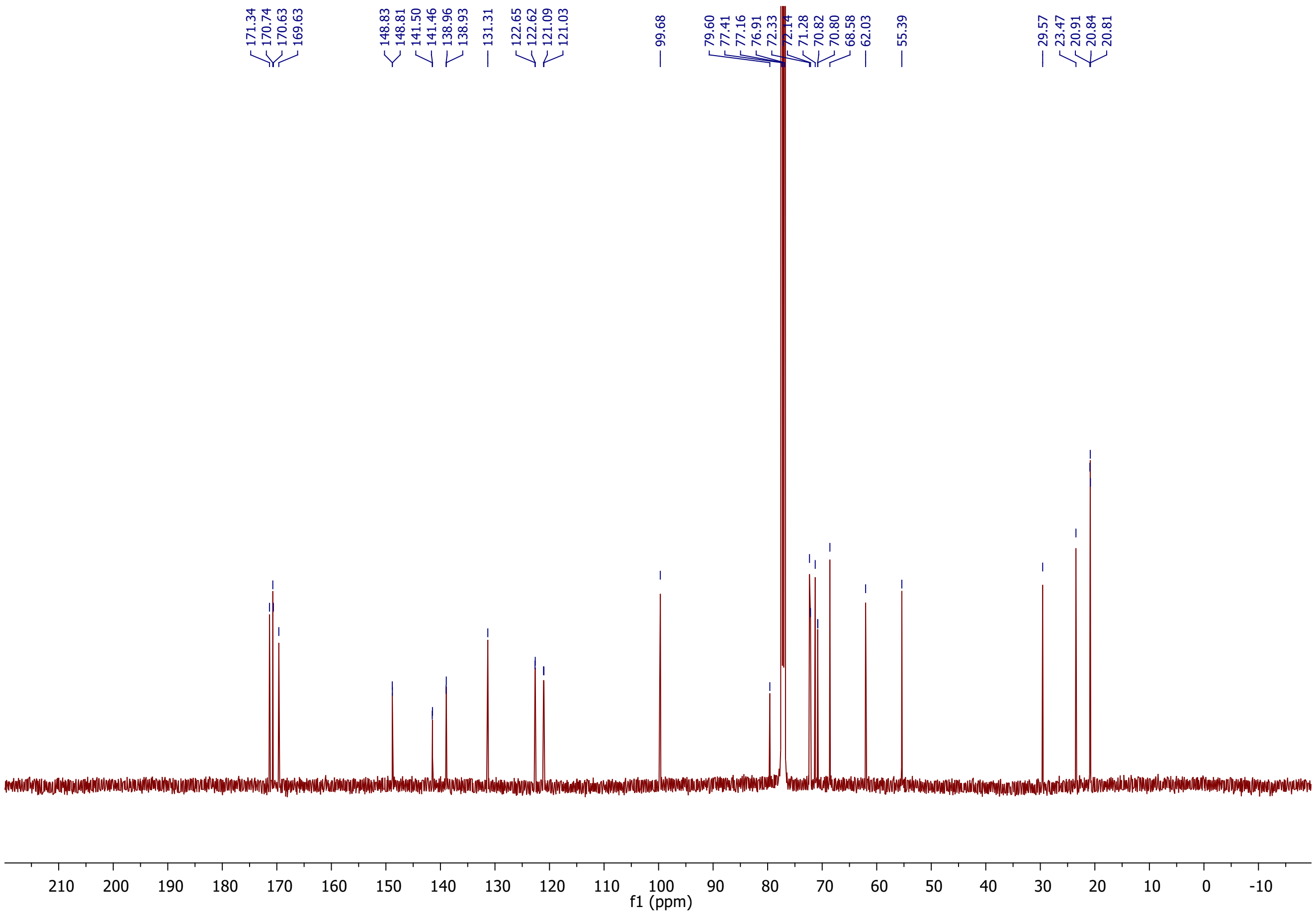


**$^{13}$C NMR spectrum (126 MHz, 298 K, $CDCl_3$) of compound 5**

Synthesis of compound 6

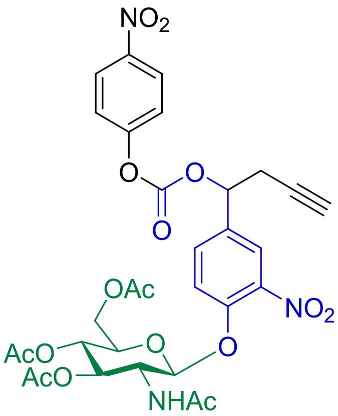


To a solution of benzyl alcohol **5** (200 mg, 0.37 mmol, 1 equiv.) solubilized in $CH_2Cl_2$ (4 mL) was added 4-nitrophenyl chloroformate (150 mg, 0.75 mmol, 2 equiv.) at room temperature. The mixture was cooled to 0°C and pyridine (75.2 µL, 0.93 mmol, 2.5 equiv.) was added. The mixture was stirred at 0°C for 20 minutes and allowed to warm to room temperature for 3 hours. After completion, the mixture was hydrolyzed with saturated $NaHCO_3$ (4 mL) and stirred for 5 minutes. Then, the organic layer was separated, and the aqueous layer was extracted with $CH_2Cl_2$ (4 mL). The combined organic layers were washed with brine (5 mL), dried over $MgSO_4$, filtrated and concentrated in *vacuo*. The crude residue was purified by chromatography on a silica gel column ($CH_2Cl_2$/MeOH 100/0 to 95/5 over 30 minutes) to yield compound **6** (188 mg, 72%) as a white solid.

**$R_f$:** 0.17 ($CH_2Cl_2$/MeOH 98/2)

**$^1$H NMR (500 MHz, $CDCl_3$, 298K):** δ ppm = 8.28 (d, *J* = 9.2 Hz, 2H), 7.91 (t, *J* = 2.3 Hz, 1H), 7.62 (dt, *J* = 8.7, 2.1 Hz, 1H), 7.42 – 7.36 (m, 3H), 5.82 – 5.78 (m, 2H), 5.68 - 5.60 (m, 2H), 5.14 (t, *J* = 9.4 Hz, 1H), 4.30 - 4.21 (m, 2H), 3.96 – 3.78 (m, 2H), 3.00 – 2.82 (m, 2H), 2.11 – 2.08 (m, 4H), 2.07 (s, 3H), 2.06 (s, 3H), 1.99 (s, 3H).

**$^{13}$C NMR (126 MHz, $CDCl_3$, 298K):** δ ppm = 171.4, 170.7, 170.5, 169.6, 155.3, 151.6, 149.9, 145.7, 141.4, 141.3, 133.4, 132.5, 132.3, 125.5, 123.8, 123.6, 121.8, 121.8, 120.8, 120.7, 99.3, 99.2, 77.4, 77.2, 76.9, 72.6, 72.4, 77.0, 68.5, 62.0, 55.7, 55.6, 26.4, 23.5, 20.9, 20.8, 20.8.

**HRMS (ESI):** $[M+Na]^+$ calcd for $C_{31}H_{31}N_3NaO_{16}$: 724.1597 found 724.1604.

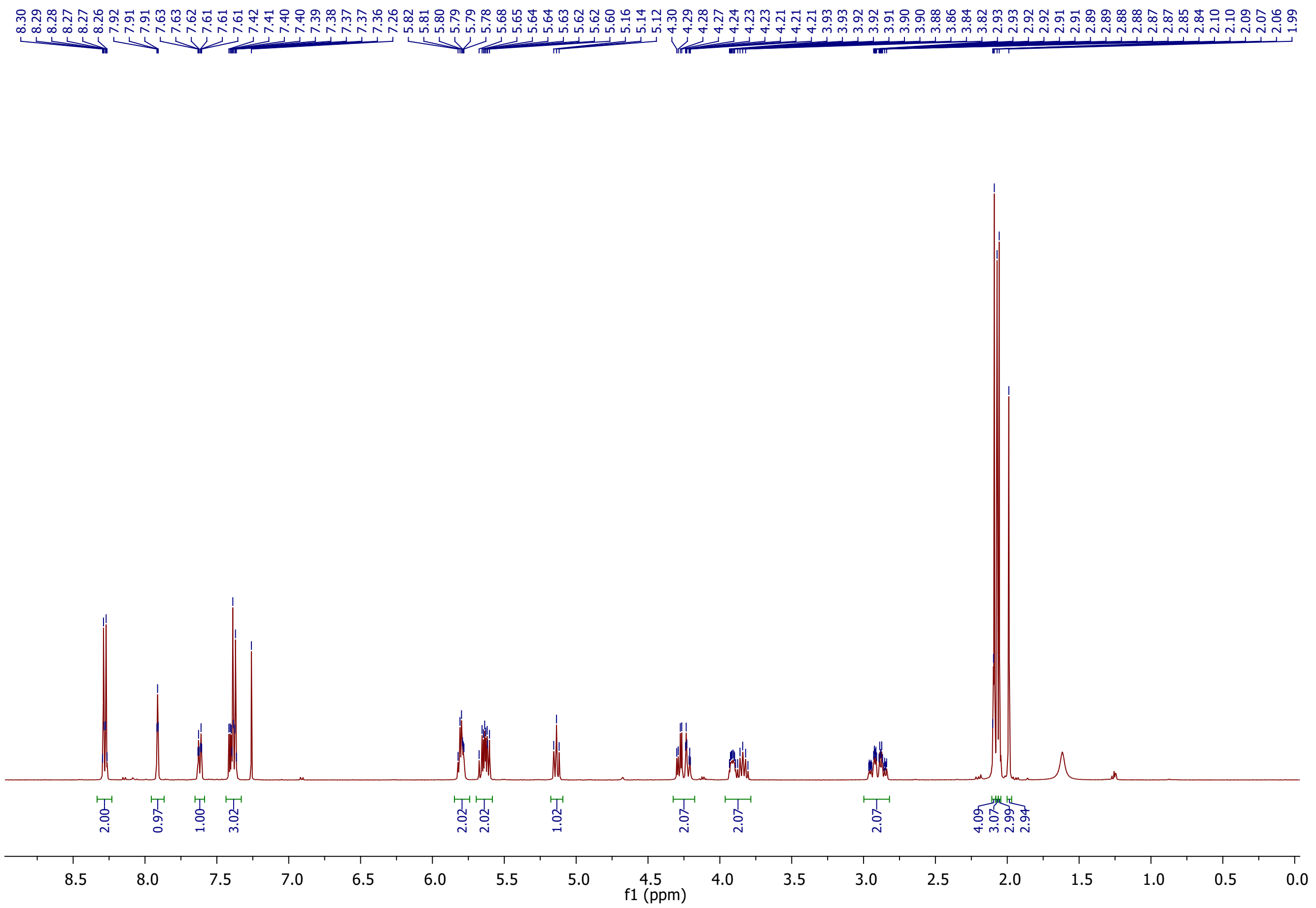

$^1H$ NMR spectrum (500 MHz, 298 K, $CDCl_3$) of compound 6

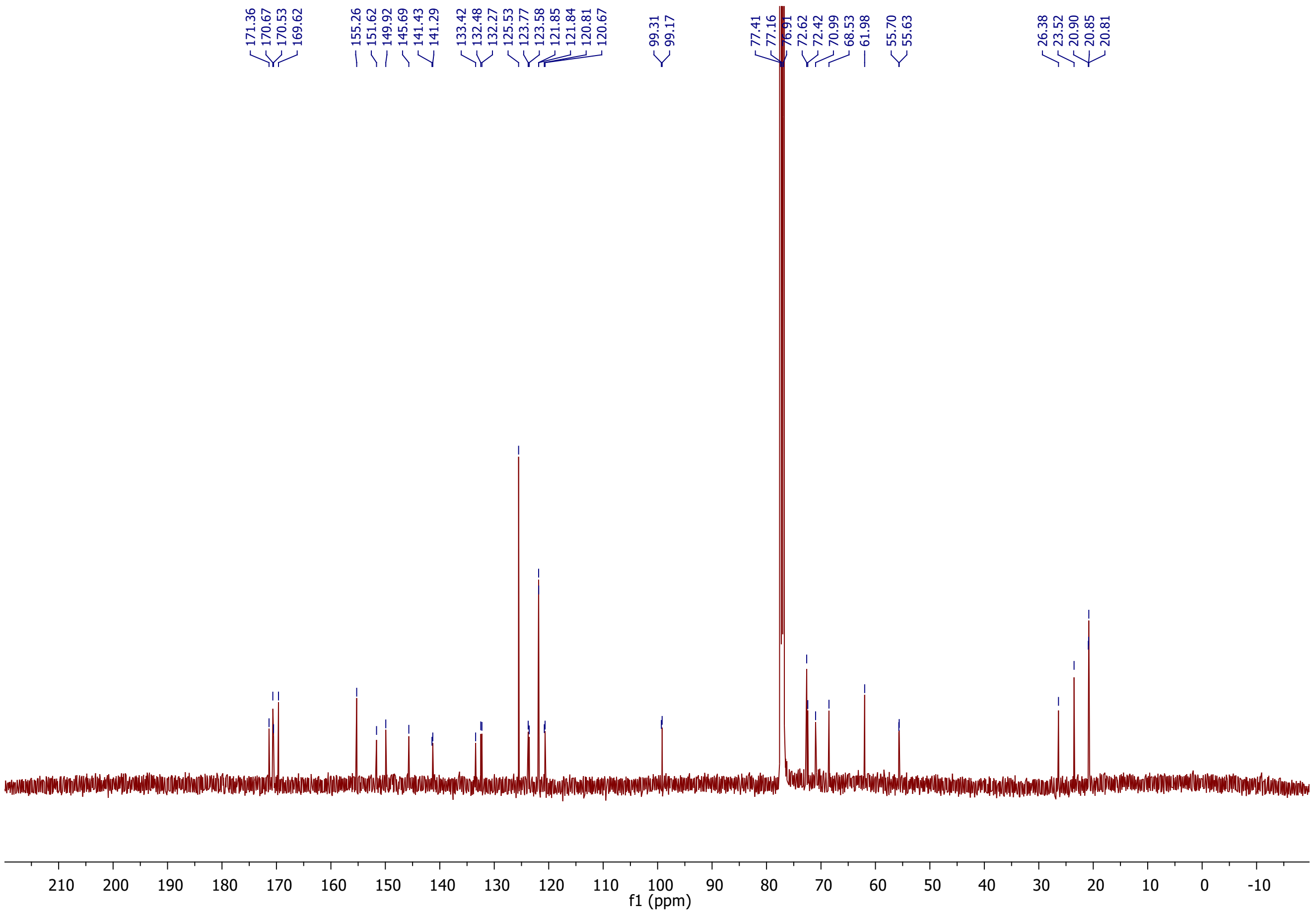


**$^{13}$C NMR spectrum (126 MHz, 298 K, $CDCl_3$) of compound 6**

Synthesis of compound 7

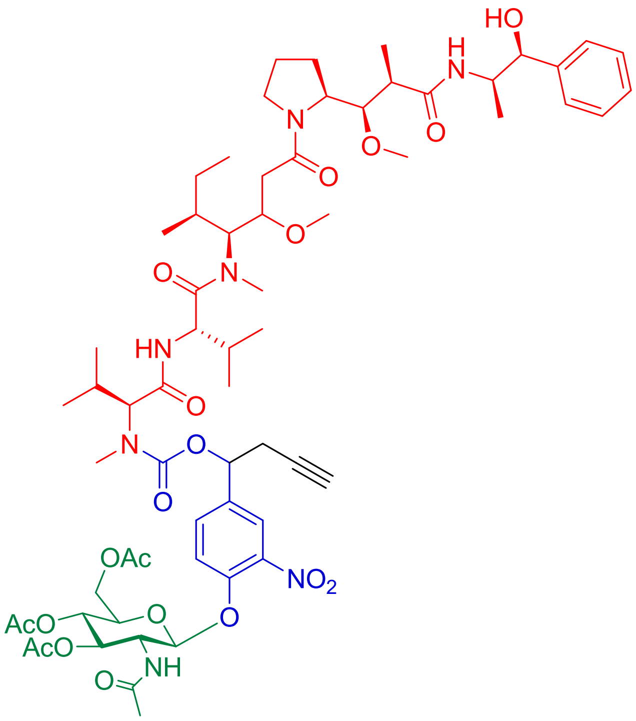


To a solution of carbonate **6** (98 mg, 0.139 mmol, 1 equiv.) solubilized in dry DMF (3 mL) were added MMAE (100 mg, 0.139 mmol, 1 equiv.) and HOBt (19 mg, 0.139 mmol, 1 equiv.). The mixture was stirred at room temperature and pyridine (0.7 mL) was added. The mixture was stirred at room temperature for 24 hours. After completion monitored by LC-MS, the solvents were evaporated under reduced pressure and the crude residue was purified by chromatography on a silica gel column ($CH_2Cl_2$/MeOH 100/0 to 90/10 over 30 minutes) to afford compound **7** (166 mg, 93%) as a white solid.

**Rt** = 10.20 min.

**MS (ESI):** $[M+H]^+$ calcd for $C_{64}H_{94}N_7O_{20}$: 1280.6 found 1280.9; $[M+2H]^{2+}$ calcd for $C_{64}H_{95}N_7O_{20}$: 640.8 found 641.3.

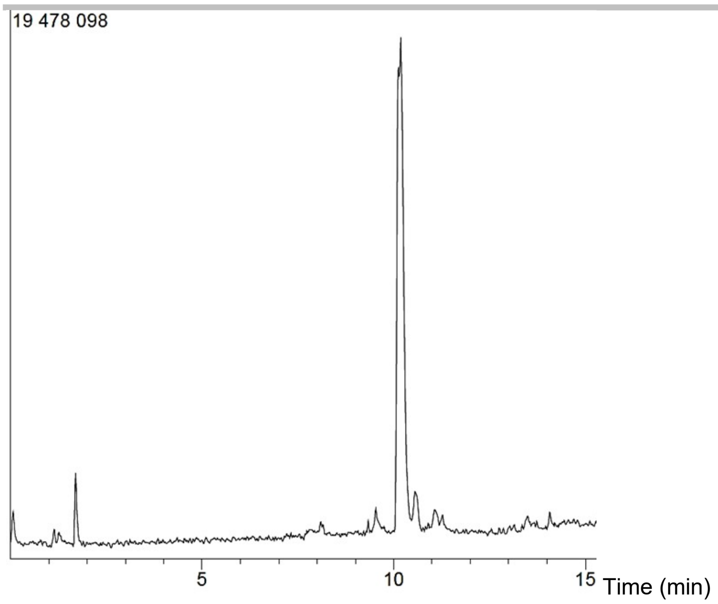


**LC-MS chromatogram of compound 7**

Synthesis of compound **1**

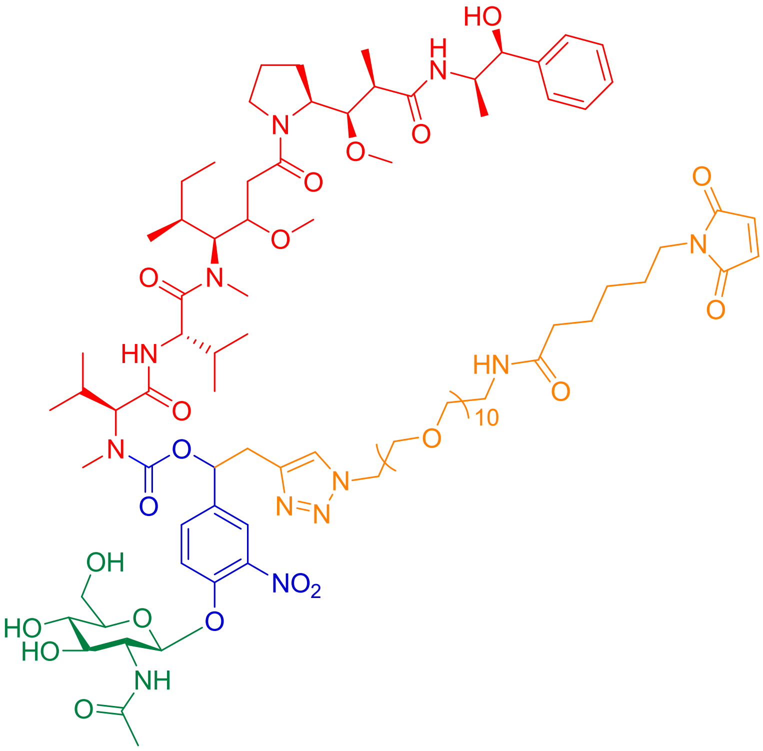

The prodrug **1** was prepared from compound **7** in 3 steps without intermediates purifications.

Firstly, to a solution of alkyne **7** (70 mg, 0.055 mmol, 1 equiv.) and azido-$PEG_{10}$-amine (32 mg, 0.060 mmol, 1.1 equiv.) in degassed $CH_2Cl_2$ (2 mL) under argon atmosphere was added $Cu(MeCN)_4PF_6$ (20 mg, 0.055 mmol, 1 equiv.). The mixture was stirred at room temperature until completion. After 3 hours, the resin QuadraPure® IDA (300 mg) was added to the mixture to scavenge the copper, stirred for additional 3 hours and removed by filtration. The solvent was evaporated under reduced pressure and the crude was used immediately for the next step without further purification.
Secondly, the crude was solubilized in MeOH (2 mL). Then, sodium methoxide (0.44 mg, 0.008 mmol, 0.15 equiv.) was added and the mixture was stirred at room temperature for 5 hours. After completion, the resin IR-120 (300 mg) was added until neutral pH. The solvent was evaporated under reduced pressure. The crude was used immediately for the next step without further purification.
Finally, $Et_3N$ (23 µL, 0.164 mmol, 3 equiv.) was added to a solution of the crude amine and 6-Maleimidocaproic acid *N*-succinimidyl ester (18.5 mg, 0.060 mmol, 1.1 equiv.) in dry DMSO (2 mL). The mixture was stirred at room temperature for 1 hour. After completion monitored by LC-MS, the solvent was evaporated under reduced pressure and the crude residue was purified by reverse phase chromatography on C18 grafted silica (gradient elution MeCN/$H_2O$ (0.05% TFA) 20/80 to 80/20 over 30 minutes) to afford **1** (25 mg, 24% over 3 steps) as a white solid.

**Rt** = 7.33 min.

**HRMS (ESI):** $[M+Na]^+$ calcd for $C_{90}H_{144}N_{12}NaO_{30}$: 1896.0004 found 1895.9952.

**$^1$H NMR (500 MHz, DMSO, 298K):** δ ppm = 8.81 – 8.62 (m, 0.25H), 8.54 – 8.37 (m, 0.5H), 8.18 – 7.99 (m, 1H), 7.97 – 7.61 (m, 6H), 7.60 – 7.49 (m, 1H), 7.48 – 7.34 (m, 1H), 7.34 – 7.22 (m, 5H), 7.20 – 7.12 (m, 1H), 7.01 (s, 2H), 5.97 – 5.73 (m, 1H), 5.48 – 5.30 (m, 1H), 5.11 – 4.94 (m, 2H), 4.86 – 4.73 (m, 1H), 4.72 – 4.63 (m, 1H), 4.61 – 4.52 (m, 1H), 4.52 – 4.28 (m, 5H), 4.22 – 4.10 (m, 1H), 4.05 – 3.91 (m, 2H), 3.80 – 3.60 (m, 6H), 3.60 – 3.54 (m, 2H), 3.46 – 3.28 (m, 34H), 3.28 – 3.10 (m, 15H), 3.09 – 2.68 (m, 5H), 2.45 – 2.39 (m, 1H), 2.29 – 2.21 (m, 1H), 2.15 – 2.08 (m, 1H), 2.02 (t, *J* = 7.4 Hz, 3H), 1.82 – 1.65 (m, 6H), 1.53 – 1.39 (m, 6H), 1.35 – 1.21 (m, 1H), 1.20 – 1.12 (m, 2H), 1.06 – 0.95 (m, 7H), 0.91 – 0.68 (m, 18H), 0.67 – 0.58 (m, 3H), 0.44 (d, *J* = 6.2 Hz, 1H).

**$^{13}$C NMR (126 MHz, DMSO, 298K):** δ ppm = 172.4, 172.3, 172.0, 171.2, 170.0, 169.6, 169.1, 168.7, 148.9, 143.7, 141.7, 140.2, 134.5, 127.9, 127.8, 126.8, 126.7, 126.4, 123.6, 99.8, 85.4, 81.6, 77.6, 74.8, 74.0, 69.8, 69.7, 69.6, 69.2, 68.9, 61.0, 60.6, 60.3, 58.7, 58.2, 57.2, 55.0, 49.8, 49.3, 46.3, 43.8, 43.2, 40.1, 40.0, 39.9, 39.9, 39.8, 39.7, 39.6, 39.5, 39.4, 39.4, 39.2, 39.0, 38.5, 37.0, 35.1, 31.6, 27.8, 25.8, 25.4, 24.8, 24.4, 23.2, 22.9, 19.0, 18.8, 15.5, 15.3, 15.1, 10.4.

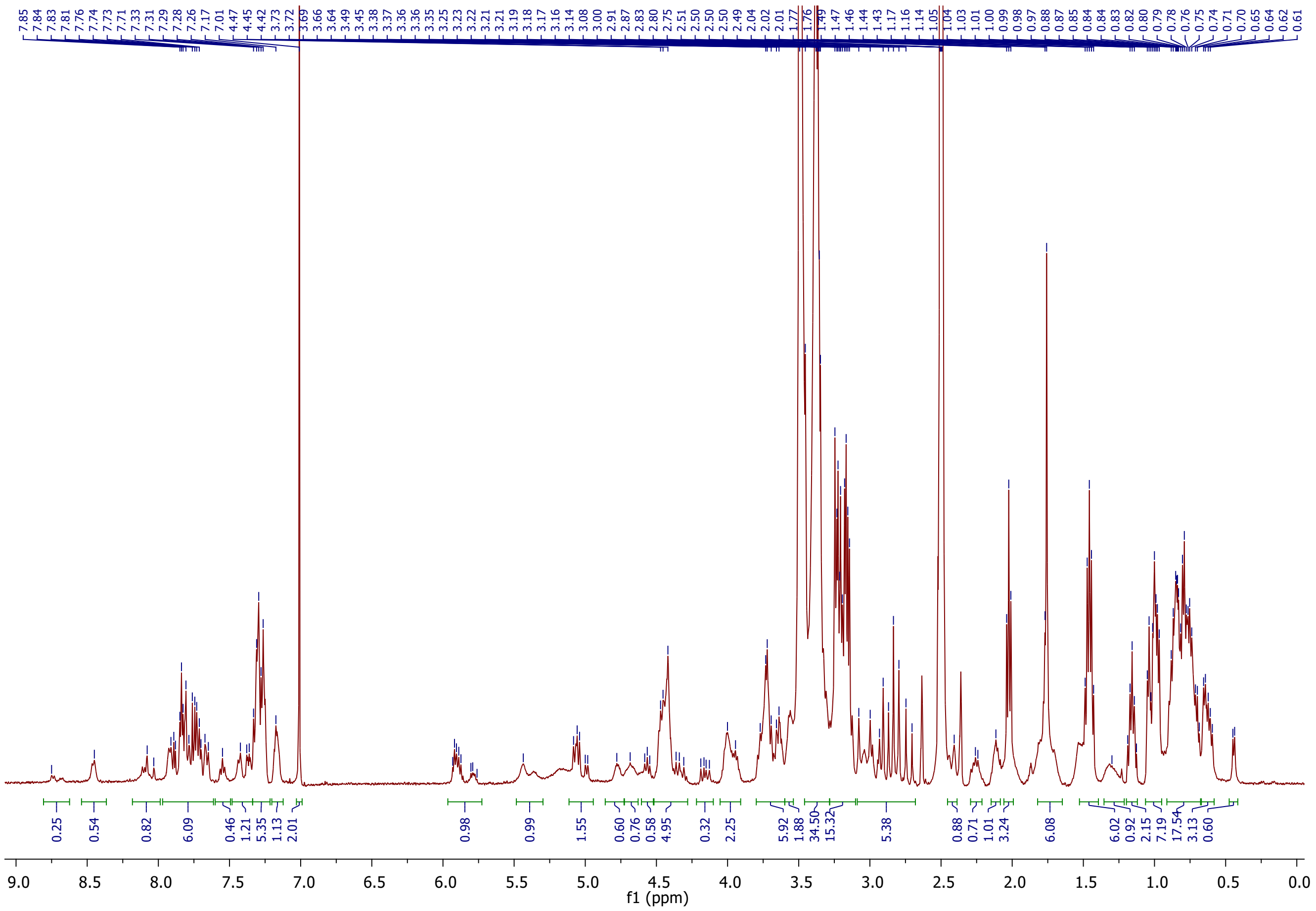


**$^{1}$H NMR spectrum (500 MHz, 298 K, DMSO-*d6*) of compound 1**

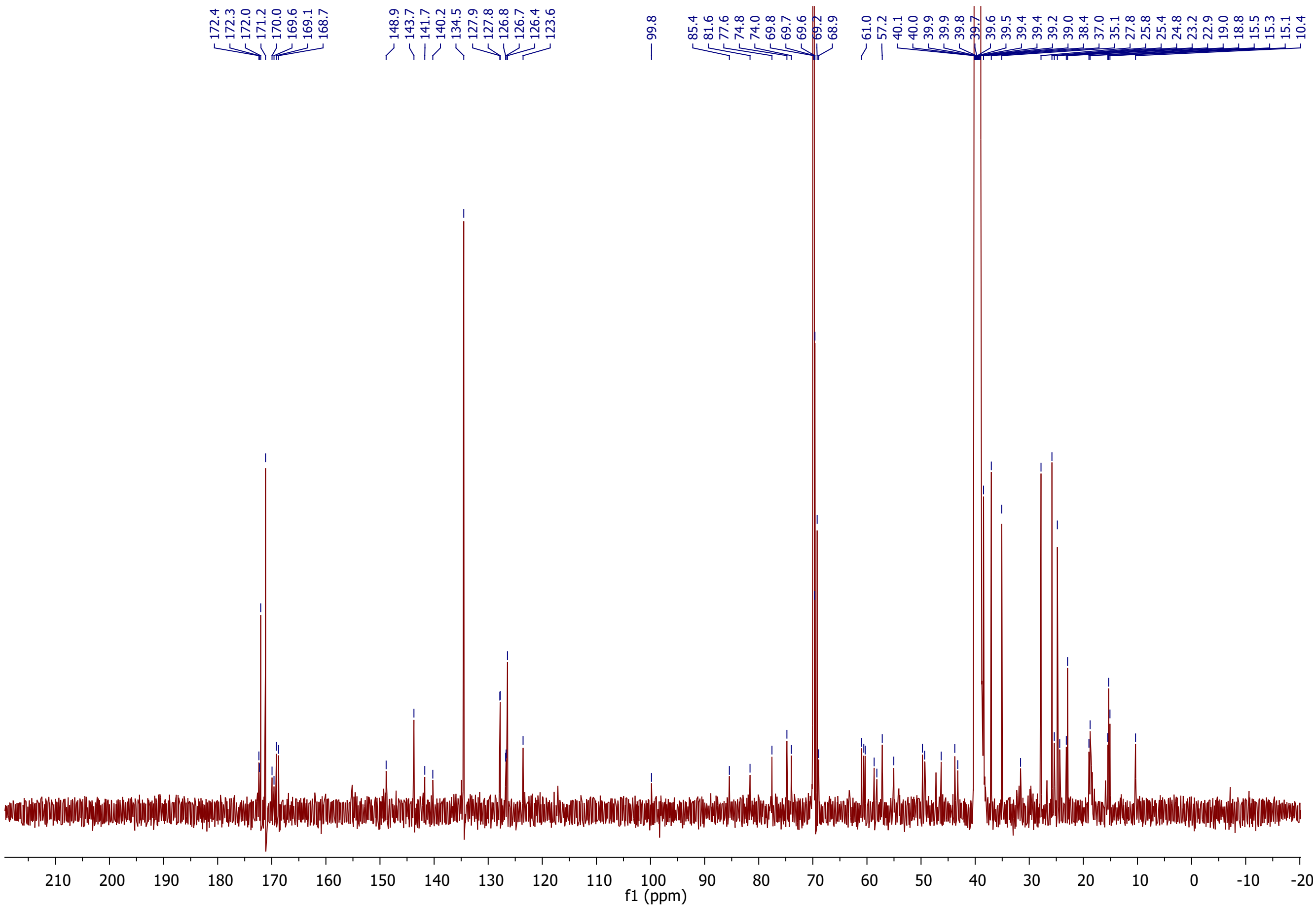


**$^{13}$C NMR spectrum (126 MHz, 298 K, DMSO-*d6*) of compound 1**

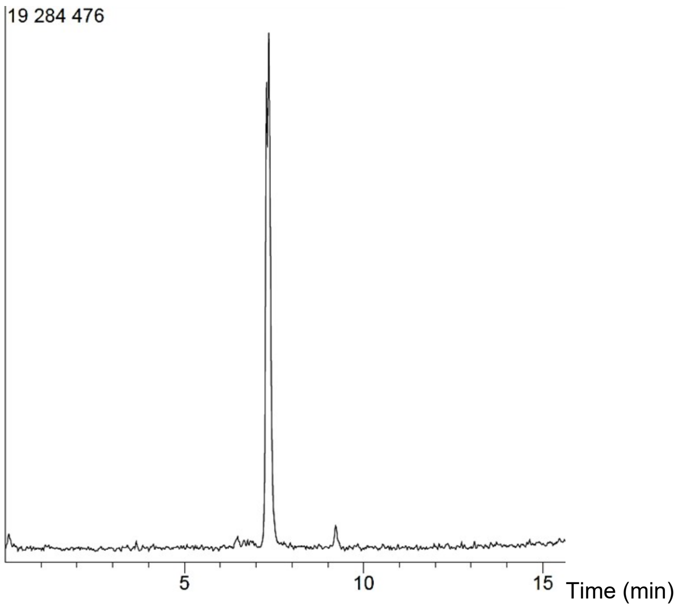


**LC-MS chromatogram of compound 1**

## 3. Trypsin-digestion experiments

Prodrug **1** (0.22 mg.mL$^{-1}$) was incubated during 4 h at 37°C in 0.02 M phosphate buffer (pH 7) containing 5% DMSO with Human Serum Albumin (23.1 mg.ml$^{-1}$, HSA, Albumin from human serum, fatty acid free, purchased from Aldrich, reference A3782). Ten microliters of this solution was diluted in 100 µL in an aqueous solution containing 50 mM of ammonium bicarbonate and 10 mM of *tris*-(hydroxymetyl)aminomethane (pH 8.5). The resulting solution was successively treated with 15 µL of a 15.4 mg.mL$^{-1}$ DL-dithiothreitol solution at 60°C for 60 min and with 15 µL of a 44.4 mg.mL$^{-1}$ iodoacetamide solution at room temperature for 45 min in the dark. The mixture was then digested overnight using a 50 µL of a 0.1 µg.mL$^{-1}$ trypsin solution . Digestion was stopped with 1 µL formic acid 99% and sample was analyzed by UHPLC/HRMS.

UHPLC/HRMS experiments were performed on an Ultimate 3000 system coupled to a Quadrupole Time of Flight (Q-TOF) mass spectrometer (Impact HD™, Bruker). An Hypersil Gold™ C18 column (50x2.1 mm, 1.9 µm) at 30°C was used for chromatographic separation at a flow rate of 0.5 mL.min$^{-1}$. The column effluent was introduced into the electrospray ionization source (ESI) of the mass spectrometer. Analyses were performed in positive ion mode. The electrospray voltage was set at 3.8 kV. The capillary was at 200°C and the dry gas (nitrogen) flow rates was set at 4 L.min$^{-1}$. Analysis of data was performed with DataAnalysis software provided by Bruker. Enzymatic hydrolysis was monitored by UHPLC/HRMS using a linear gradient composed of A (0.1% formic acid in water) and B (0.1% formic acid in $CH_3CN$) (method 3). After injection of 10 µL of sample, the gradient started with 95% of A. It remained constant during 3 min and decreased to 35% in 11 min. The gradient finally reached 10% of A in 1 min. The column was then reconditioned for 3 min with 95 % of A.

The formation of the coupling product was confirmed by the detection of the HSA peptide including the cysteine 34 linked to **1** (m/z 1077.3241 $[M+4H]^{4+}$ and m/z 1436.0964 $[M+3H]^{3+}$, Figure S4).

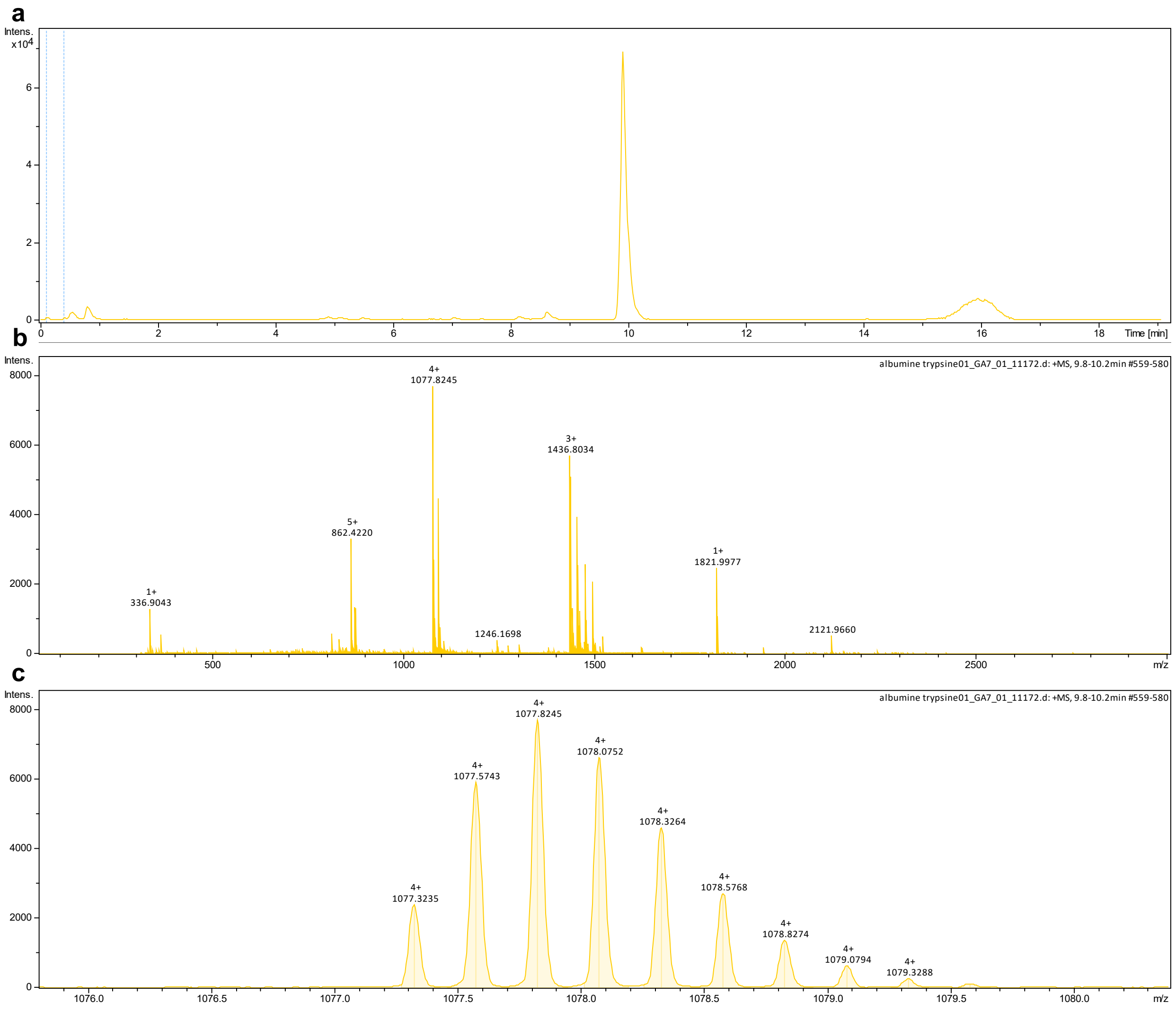

d

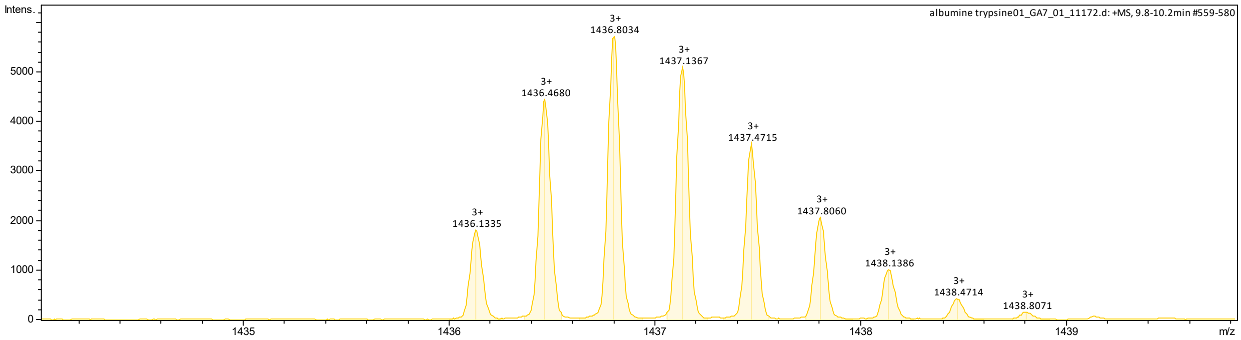


**Figure S4.** UHPLC-HRMS of trypsin digest of HSA incubated with 1. a. Chromatographic peak of the ion corresponding to HSA peptide including the cysteine 34 linked to the prodrug 1**.** b. MS spectrum of this ion. c and d. MS isotopic patterns of the coupling product four and three charge state (m/z 1077.3241 $[M+4H]^{4+}$ and m/z 1436.0964 $[M+3H]^{3+}$)**.**

## 4. Stability and enzymatic cleavage

Prodrug **1** (0.33 mg.mL$^{-1}$) was incubated at 37°C in 0.02 M phosphate buffer (pH 7) containing 5% DMSO with Human Serum Albumin (46.8 mg.ml$^{-1}$, HSA, Albumin from human serum, fatty acid free, purchased from Aldrich, reference A3782) for 3 hours to form the corresponding bioconjugate through Michael addition. The evolution of the mixture was monitored by HPLC-UV (λ = 254 nm). The mixture was then diluted 1:10 using 0.02 M phosphate buffer (pH 7) and *N*-acetyl-β-D-glucosaminidase, from Canavalia ensiformis (0.227 U.mL$^{-1}$; purchased from Sigma Adrich, reference A2264) was added at 37°C. The release of MMAE along the time was followed by LC-MS (screened MMAE m/z: 718 $[M+H]^{+}$).

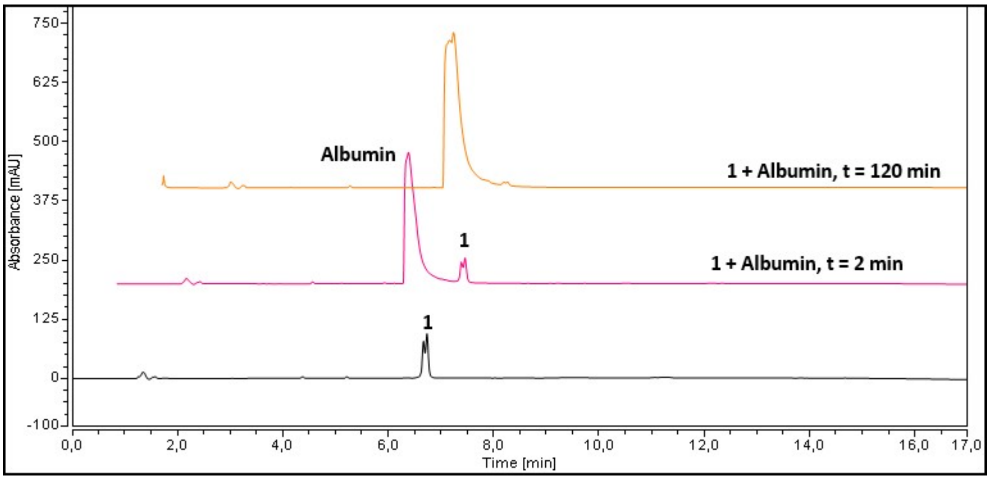


**Figure S5.** Binding of **1** with HSA over the time recorded by HPLC-UV.

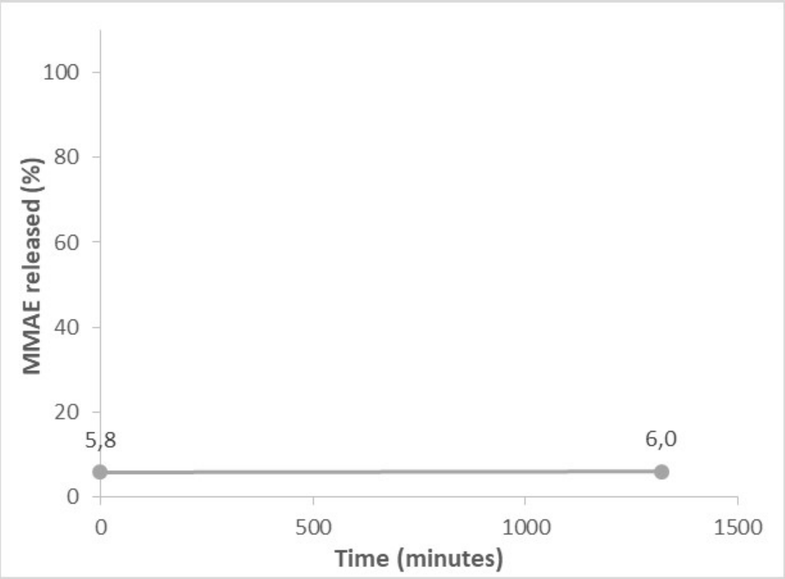


**Figure S6**. Release of MMAE from prodrug **1** bound to albumin over time in the absence of *N*-acetyl-β-D-glucosaminidase recorded by LC-MS.

# III. Biological evaluation of prodrug 1

## 1. Cell viability

MDA-MB-231 human breast cancer cell line was obtained from Perkin Elmer. The Cell Proliferation Kit II XTT was used to measure cell viability. $3x10^3$ MDA-MB-231 cells/well were seeded in a 96-well plate, in RPMI 1640-GlutaMAX supplemented by 10 % foetal bovine serum and 1 % Penicillin/Streptomycin. Twenty-four hours later, cells were exposed to MMAE or **1** in the presence or absence of β-GlcNAc (4 $U.well^{-1}$). After 3 days of treatment, 25 µl of the XTT labelling mixture were added per well. After additional 4 h of incubation, absorbance was determined at 490 nm on a Berthold Mithras 96-well microplate reader. Experiments were performed 3 times in triplicate. Data were analysed with GraphPad software.

The whole experiment was repeated with the galactoside (Gal-MMAE) and glucuronide (Glu-MMAE) analogues of **1** (Figure S7a) in the presence of β-Gal or β-Glu from *Escherichia coli* (Sigma Aldrich, Mannheim, Germany) respectively (4 $U.well^{-1}$). For each compound, inhibitory concentration values ($IC_{50}$) were determined with GraphPad software (Figure S7b).

**a**

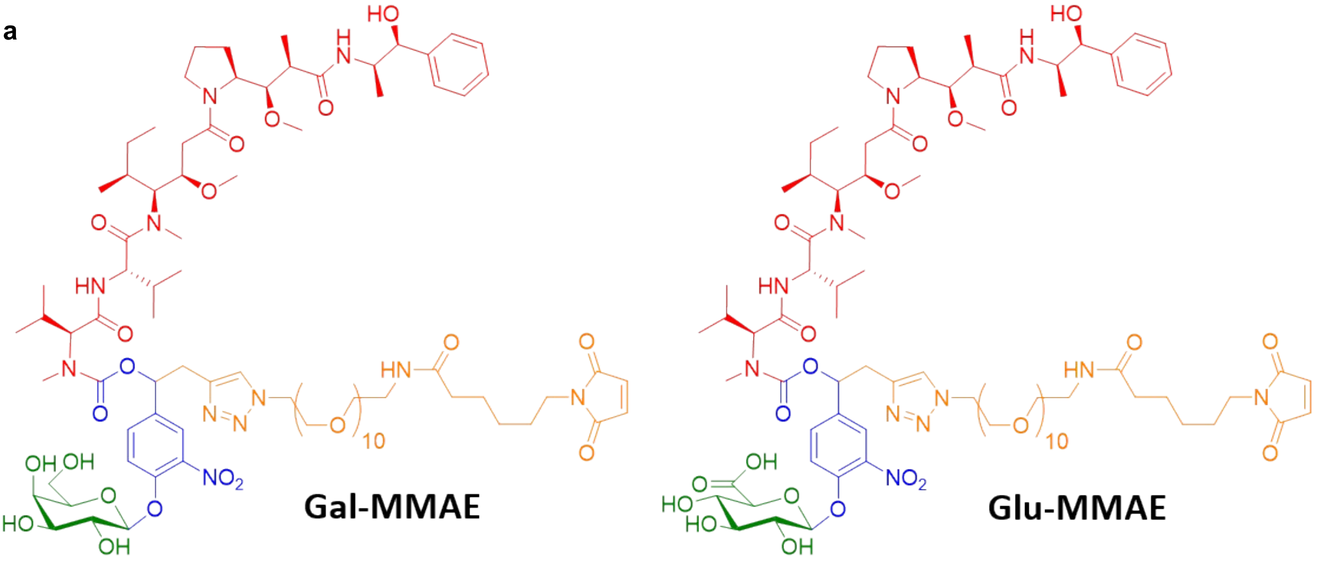

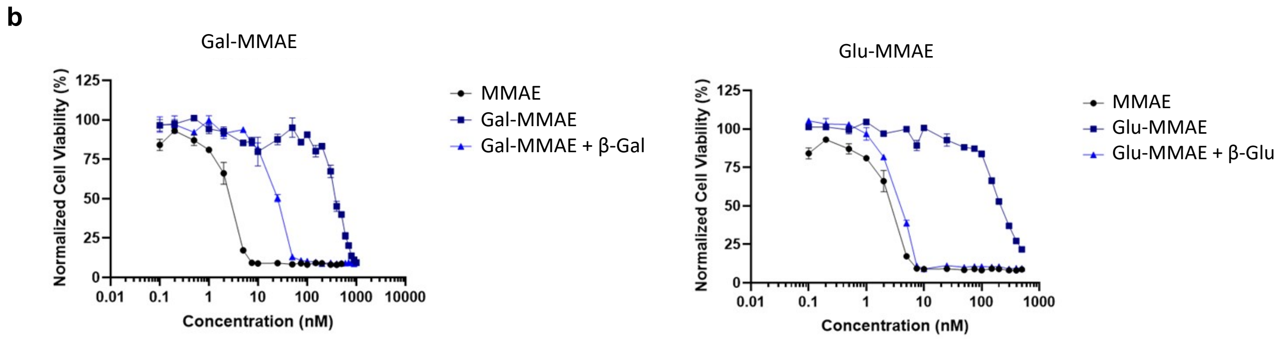


| | $IC_{50}$ (nM) |
|---|---|
| Gal-MMAE | 3294 |
| Gal-MMAE + β-Gal | 20.2 |

| | $IC_{50}$ (nM) |
|---|---|
| Glu-MMAE | 772 |
| Glu-MMAE + β-Glu | 2.3 |

**Figure S7**. a. Structure of the galactoside (Gal-MMAE) and glucuronide (Glu-MMAE) analogues of prodrug **1**. b. Antiproliferative activities and $IC_{50}$ of Gal-MMAE and Glu-MMAE with or without β-Gal and β-Glu, respectively, after 3 days treatment. Each point shows mean ± s.e.m from 3 experiments.

## 2. *In vivo* experiments

Female, 6- to 8-week-old Nude mice were purchased from Charles River Laboratories. Mice were acclimated for 7 days in the laboratory before experimentation and were maintained in sterilized filter-stopped cages inside a controlled ventilated rack and had access to food and water *ad libitum*. The experimental procedure involving animals was validated by the regional ethical comity (CECCO n°3) and carried out in accordance with the guidelines of the French Agriculture and Forestry Ministry (decree 2013-118) and of the European Communities Council Directive (2010/63/UE). All along the studies, mice were examined at least 3 times a week for clinical signs, distress, decreased physical activity and body weight as indicators of the health status.

Human breast cancer xenografts from the MDA-MB-231-luc breast cancer cell line were established in BALB/c Nude mice by orthotopic implantation. Mice were anaesthetised by inhalation of 1.5% isoflurane with air. The inoculum ($2x10^6$ tumour cells in 100 µL PBS) was injected in the mammary fat pads of animals. Mice received treatment 21 days after tumour implantations. Two groups were designed and received once every two weeks intravenous injections of a 5% DMSO and 95% PBS mix (vehicle group) or 4 mg/kg of compound **1** (days 21, 35, 49, n = 6). Tumour volumes were determined by ultrasound imaging once a week during 6 weeks after treatment initiation.

## References

(1) Lange, J.; Eddhif, B.; Tarighi, M.; Garandeau, T.; Péraudeau, E.; Clarhaut, J.; Renoux, B.; Papot, S.; Poinot, P. Volatile Organic Compound (VOC)-Based Probe for Induced Volatolomics of Cancers. *Angew. Chem. Int. Ed.* **2019**, *58* (49), 17563–17566. https://doi.org/10.1002/anie.201906261.

(2) Carlsson, G.; Gullberg, B.; Hafström, L. Estimation of Liver Tumor Volume Using Different Formulas—An Experimental Study in Rats. *J. Cancer Res. Clin. Oncol.* **1983**, *105* (1), 20–23. https://doi.org/10.1007/BF00391826.

(3) Zhang, J.; Liu, Z.; Zhong, S.; Wang, H.; Caidan, B.; Yin, H.; Dang, Z. Strategy for Effective Inhibition of Arylsulfatase/β-Glucuronidase to Prevent Deconjugation of Sulfate and Glucuronide Conjugates in Wastewater during Sample Collection and Storage. *Sci. Total Environ.* **2020**, *703*, 135536. https://doi.org/10.1016/j.scitotenv.2019.135536.

(4) Beratis, N. G.; Georgiou, G.; Eliopoulou, M. Increased Activity of Lysosomal Enzymes in the Peritoneal Fluid of Bacterial Peritonitis. *Pediatrics* **2002**, *109* (3), e44–e44. https://doi.org/10.1542/peds.109.3.e44.

(5) Thomas, M.; Clarhaut, J.; Strale, P.-O.; Tranoy-Opalinski, I.; Roche, J.; Papot, S. A Galactosidase-Responsive “Trojan Horse” for the Selective Targeting of Folate Receptor-Positive Tumor Cells. *ChemMedChem* **2011**, *6* (6), 1006–1010. https://doi.org/10.1002/cmdc.201100114.
